# Heme drives cardiac endothelial senescence in sepsis via STING activation

**DOI:** 10.1038/s41419-025-08370-w

**Published:** 2025-12-18

**Authors:** Tingting Li, Peilin Zhu, Jialing Wang, Tao Zhang, Joseph Adams, Fei Tu, Chloe Garbe, Xiaojin Zhang, Li Liu, Krishna Singh, David L. Williams, Chuanfu Li, Xiaohui Wang

**Affiliations:** 1 Department of Biomedical Sciences, Quillen College of Medicine, East Tennessee State University, Johnson City, TN USA; 2 UMPC Hillman Cancer Center, University of Pittsburgh, Pittsburgh, PA USA; 3 Department of Geriatrics, Jiangsu Provincial Key Laboratory of Geriatrics, The First Affiliated Hospital of Nanjing Medical University, Nanjing, China; 4 Center of Excellence in Inflammation, Infectious Disease, and Immunity, Quillen College of Medicine, East Tennessee State University, Johnson City, TN USA; 5 Department of Surgery, Quillen College of Medicine, East Tennessee State University, Johnson City, TN USA

**Keywords:** Cardiomyopathies, Senescence

## Abstract

Sepsis-induced cardiac dysfunction is a major contributor to sepsis-related mortality, and many patients continue to experience long-term cardiac complications after recovery. Here, we demonstrate that cardiac senescence is a key feature of sepsis-associated cardiac dysfunction, with endothelial cells identified as the predominant senescent population in septic cardiac tissue. However, the pathogenic drivers of endothelial senescence in sepsis remain poorly characterized. Among potential mediators, we found that elevated levels of heme, a byproduct of hemolysis, strongly correlate with increased endothelial senescence and impaired cardiac function. Mechanistic studies revealed that heme acts as a novel ligand for STING, exacerbating bacterial infection-induced STING polymerization and activation, thereby promoting endothelial senescence. Notably, either STING inhibition or enhanced heme clearance via increased hemopexin expression significantly alleviated cardiac endothelial senescence and facilitated cardiac functional recovery in septic mice. These findings identify heme as a critical pathogenic driver of endothelial senescence and highlight heme clearance as a promising therapeutic strategy for mitigating sepsis-induced cardiac dysfunction.

## Introduction

Sepsis, a life-threatening condition characterized by a dysregulated immune response to infection, remains a leading cause of mortality in intensive care units worldwide, accounting for approximately 20% of all global deaths [1, 2]. Among the various organs affected in sepsis, the heart is especially susceptible to injury [3–5]. Sepsis-induced cardiomyopathy is a major contributor to poor outcomes, with many septic patients experiencing long-term cardiac complications. However, its underlying pathophysiological mechanisms remain poorly defined, and no targeted therapies are currently available.

Endothelial cells, which constitute the largest organ in the body, are essential for maintaining vascular integrity, regulating immune responses, and supporting metabolic homeostasis [6–8]. Endothelial dysfunction is a hallmark of sepsis and a critical driver of disease progression [9–12]. In the heart, endothelial injury disrupts tissue oxygenation, increases vascular leakage, and exacerbates inflammation, thereby contributing to myocardial dysfunction [10, 12]. Although mechanisms such as cytokine release, oxidative stress, coagulopathy, and glycocalyx degradation have been implicated in septic endothelial injury [11–13], the factors driving cardiac endothelial dysfunction during sepsis remain largely undefined.

Another important yet underexplored mechanism that may contribute to septic endothelial injury is cellular senescence. Cellular senescence, characterized as a state of cell cycle arrest and the acquisition of a proinflammatory senescence-associated secretory phenotype (SASP), has emerged as a critical contributor to endothelial dysfunction in various cardiovascular diseases [14–18]. Senescent endothelial cells lose their regenerative capacity, promote chronic inflammation, and impair vascular repair [15, 16, 19–21]. Although recent studies suggest that sepsis is associated with endothelial cell loss and reduced proliferative capacity, it remains unclear whether sepsis actively induces endothelial senescence and whether this process impairs cardiac functional recovery and contributes to the development of sepsis-associated cardiomyopathy.

Hemolysis is a common feature of sepsis and is strongly associated with disease severity and poor clinical outcomes [22–24]. During hemolysis, the breakdown of red blood cells leads to the release of free heme into the circulation [25]. Free heme is now recognized as a potent danger-associated molecular pattern (DAMP) that promotes oxidative stress, amplifies inflammation, and induces cell death [22, 24, 26–29]. However, whether free heme contributes to endothelial dysfunction and cardiac impairment during sepsis remains unclear.

In the present study, we identify pronounced cardiac endothelial senescence as a hallmark of sepsis. Senescence levels correlated with disease severity and were associated with impaired vascular repair capacity and adverse outcomes. We further demonstrate that free heme, released during hemolysis in sepsis, acts as a key mediator of cardiac endothelial senescence and dysfunction. Elevated heme levels are strongly associated with impaired cardiac function and increased mortality. Although our recent work showed that heme contributes to Kupffer cell senescence via cGAS-STING activation, the mechanisms by which heme activates this pathway remained unclear [22]. In this study, we provide mechanistic evidence that heme functions as a novel ligand of STING, promoting its oligomerization and subsequently amplifying STING-TBK1 activation, a known trigger of endothelial senescence [30–32]. Importantly, pharmacological inhibition of STING effectively mitigated endothelial senescence and improved cardiac functional recovery in septic mice. Furthermore, therapeutic upregulation of hemopexin, a heme scavenger [22, 23], significantly reduced circulating free heme levels, alleviated cardiac endothelial senescence, and enhanced cardiac functional recovery during sepsis.

Collectively, these findings uncover a novel role of heme in driving cGAS–STING–mediated endothelial senescence and cardiac dysfunction during sepsis and highlight heme clearance as a promising therapeutic strategy to improve sepsis outcomes.

## Results

### Sepsis induces cardiac senescence that correlates with disease severity

**Fig. 1:**
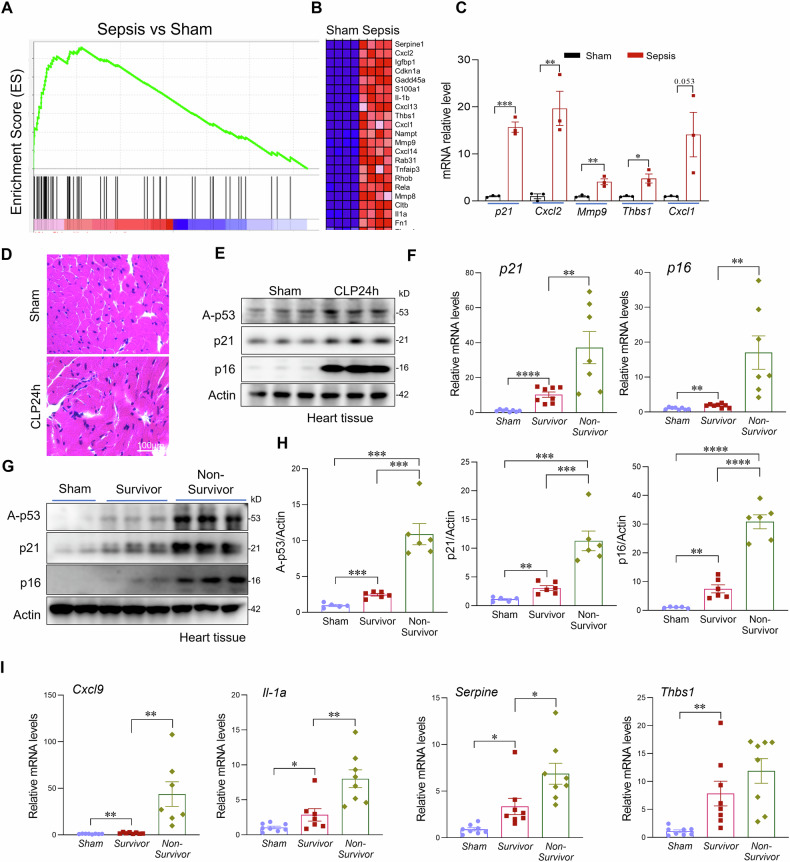
Sepsis induces cardiac senescence that correlates with disease severity. **A** Gene set enrichment analysis (GSEA) of RNA-seq data from hearts of sham and septic mice. **B** Heat map showing differential expression of selected senescence-associated genes. **C** Expression levels of representative senescence-related genes (Cdkn1a/p21, Cxcl2, Mmp9, Thbs1, Cxcl1) based on RNA-seq analysis of hearts from sham and septic mice. **D** H&E staining of sham and septic heart tissues. Scale bars, 100 μm. **E** Western blot analysis of senescence markers p16, p21, and acetylated p53 in sham and septic heart tissues (n = 6 mice/group). **F** qRT-PCR analysis of senescence markers in sham and septic heart tissues (n = 6–8 mice/group). **G**, **H** Western blot analysis and quantification of senescence markers p16, p21, and acetylated p53 in heart tissues from sham mice, septic non-survivors (body temperature < 30 °C), and septic survivors (>30 °C) at 24 h post-CLP (n = 6 mice/group). **I** qRT-PCR analysis of senescence associated genes (Cxcl9, Il-1A, Serpine1, Thbs1) in heart tissues from sham mice, septic survivors, and septic non-survivors. All data are presented as mean ± SD. *P < 0.05, **P < 0.01, ***P < 0.001, ****P < 0.0001.

Given the critical role of cellular senescence in cardiovascular pathology and its potential contribution to sepsis-induced cardiac dysfunction [16, 33–35], we first examined whether sepsis promotes cardiac senescence. Sepsis was induced in mice using the cecal ligation and puncture (CLP) model, and heart tissues were collected 24 h post-sepsis for analysis. Gene set enrichment analysis (GSEA) of RNA sequencing (RNA-seq) data revealed a significant upregulation of gene signatures associated with cellular senescence in septic hearts (Fig. 1A–C). Hematoxylin and eosin (H&E) staining revealed disorganized myocardial architecture and inflammatory cell infiltration in septic hearts, indicative of acute tissue injury (Fig. 1D). Notably, senescence-associated secretory phenotype (SASP) markers, including MMPs, IL-1α, IL-1β, and CXCL family cytokines, as well as the cell cycle inhibitors p21 and p16, were markedly increased in septic hearts compared to sham controls (Fig. 1A–C). Consistent with transcriptomic findings, western blot analysis showed a substantial increase in protein levels of key senescence markers, including p21, p16, and acetylated p53 (Ac-p53), in heart tissues from septic mice (Fig. 1E) [36]. To investigate whether cardiac senescence correlates with sepsis severity, we compared the expression of senescence markers between survivors and non-survivors. Since a body temperature below 30 °C is a reliable predictor of mortality in septic mice, we stratified mice at 24 h post-CLP into survivors (body temperature >30 °C) and non-survivors (body temperature <30 °C) [37]. qPCR analysis revealed significantly higher expression of p21, p16, and SASP factors in non-survivors compared with survivors (Fig. 1F–I). Together, these results provide compelling evidence that sepsis induces severe cardiac senescence, which correlates with sepsis severity.

### Cardiac endothelial cells are the predominant senescent population during sepsis

**Fig. 2:**
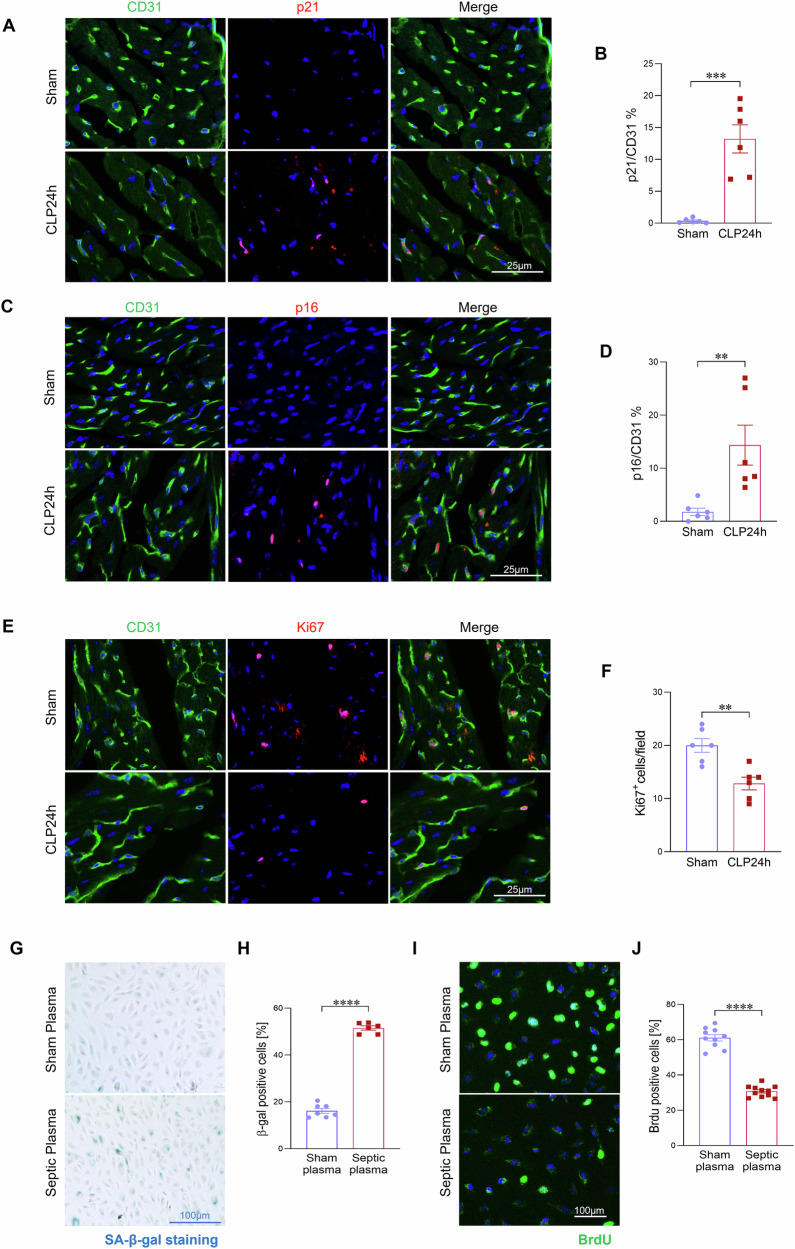
Cardiac endothelial cells are the predominant senescent population during sepsis. **A**–**D** Co-immunostaining of senescence markers p21 and p16 with the endothelial cell marker CD31, and their quantification in heart sections from sham and septic mice (n = 6 mice/group). Scale bars, 25 μm. **E**, **F** Co-immunostaining of the cellular proliferation marker Ki67 with the endothelial marker CD31 in heart tissues from sham controls and septic mice at 24 h post-CLP, along with corresponding quantification. (n = 8/group). Scale bars, 25 μm. **G**, **H** β-gal staining and quantification of HUVECs treated with plasma (1:200) from sham or septic (CLP 24 h) mice for 24 h (n = 6/group). Scale bars, 100 μm. **I**, **J** BrdU incorporation assay and quantification of HUVECs from the indicated treatment groups (n = 6/group). Scale bars, 100 μm. All data are presented as mean ± SD. *P < 0.05, **P < 0.01, ***P < 0.001, ****P < 0.0001.

To identify the primary senescent cell population in the septic heart, we performed co-immunostaining of the senescence markers p21 and p16 with CD31, an established endothelial cell marker. Quantitative analysis showed a ~30-fold increase in p21-positive endothelial cells and a ~16-fold increase in p16-positive endothelial cells in septic heart tissues compared with sham controls (Fig. 2A–D). Furthermore, p21 and p16 staining largely co-localized with CD31 (Fig. 2A–D), indicating that endothelial cells are the predominant senescent population in the septic heart. To investigate whether cardiac endothelial senescence correlates with sepsis severity, we compared senescence marker expression between survivors and non-survivors at 24 h post-CLP. As shown in Fig. S1A–D, non-survivors exhibited significantly higher levels of endothelial senescence, as evidenced by an increased number of p21 and p16 positive endothelial cells, compared to survivors (Fig. S1A–D). In addition, we observed that cardiac endothelial density was significantly reduced after sepsis (Fig. S2A). TUNEL staining revealed markedly increased endothelial apoptosis, particularly in non-survivors (Fig. S2D–E), suggesting that apoptosis contributes to endothelial loss. Because senescence impairs endothelial proliferation needed for vascular repair, we then assessed endothelial proliferation. Ki67 immunostaining revealed a pronounced reduction in endothelial proliferation in septic hearts, with further decreases in non-survivors (Figs. 2E, F and S2A–E).

To further determine whether circulating factors in sepsis promote endothelial cell senescence, human umbilical vein endothelial cells (HUVECs) were exposed to plasma from septic or sham mice for 24 h. Cells treated with septic plasma showed a significantly greater number of β-galactosidase-positive cells compared with controls, indicating the presence of senescence-inducing factors in the plasma (Fig. 2G, H). In parallel, BrdU incorporation assays showed reduced endothelial proliferation following exposure to septic plasma, supporting the idea that sepsis-associated circulating mediators directly compromise endothelial proliferation (Fig. 2I, J). Together, these findings demonstrate that cardiac endothelial cells are the main senescent population in sepsis, showing high susceptibility to systemic inflammatory stress and impaired proliferation. This dysfunction may compromise vascular repair and contribute to organ damage, highlighting endothelial senescence as a potential therapeutic target in sepsis.

### Elevated heme levels correlate with cardiac endothelial senescence and dysfunction in sepsis

**Fig. 3:**
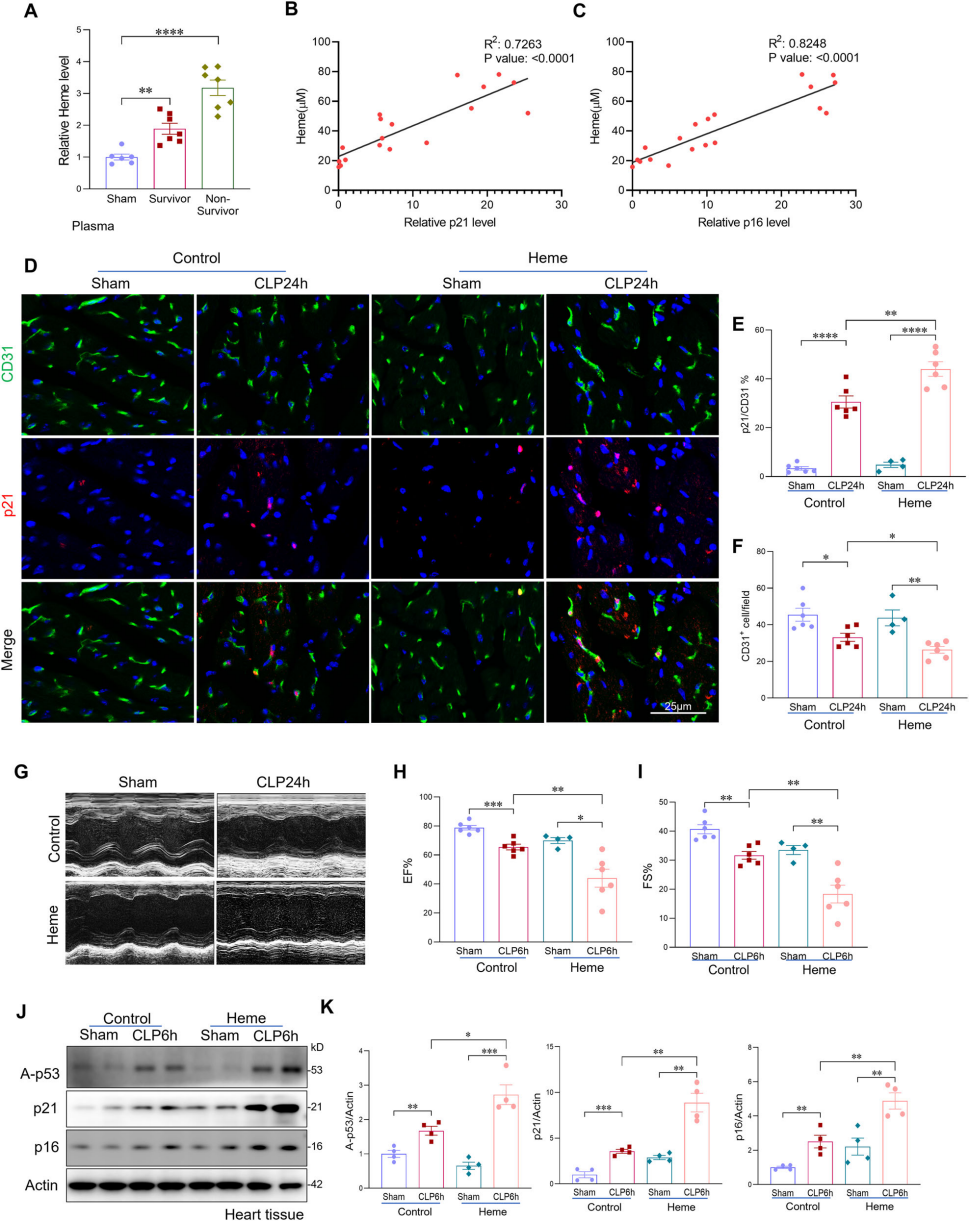
Elevated heme levels correlate with cardiac endothelial senescence and dysfunction in sepsis. **A** Relative plasma heme levels in sham mice, septic non-survivors (body temperature <30 °C), and septic survivors (>30°C) at 24 h post-CLP (n = 6–8 mice/group). **B**, **C** Mice with higher circulating heme levels exhibited increased cardiac endothelial senescence, as indicated by elevated expression of senescence markers p21 and p16. **D**, **E** Co-immunostaining of p21 with endothelial cell marker CD31 and quantification in heart tissues from sham and septic mice with or without heme treatment (n = 6 mice/group). Scale bars, 25 μm. **F** Quantification of the endothelial cell marker CD31 in heart tissues from sham and septic mice with or without heme treatment (n = 6 mice/group). **G**–**I** Echocardiographic assessment of ejection fraction (EF) and fractional shortening (FS) in sham and septic mice treated with or without heme (n = 6 mice/group). **J**, **K** Western blot analysis and quantification of senescence markers p16, p21, and acetylated p53 in heart tissues from sham and septic mice with or without heme treatment (n = 6 mice/group). All data are presented as mean ± SD. *P < 0.05, **P < 0.01, ***P < 0.001, ****P < 0.0001.

Hemolysis and the subsequent release of free heme are well-recognized complications in sepsis [22, 23, 25]. Considering the association between elevated heme levels and sepsis severity, we investigated whether circulating heme contributes to sepsis-induced endothelial cell senescence. To assess this, we first measured circulating free heme levels and examined their correlation with cardiac endothelial senescence in septic mice (Fig. 3A–C). As shown in Fig. 3B, C, septic mice with higher plasma heme concentrations exhibited a marked increase in endothelial cell senescence compared with those with lower heme levels. This finding reveals a positive correlation between circulating heme levels and the severity of cardiac endothelial senescence. To further elucidate the causal role of heme in endothelial senescence during sepsis, we administered exogenous heme (15 mg/kg, IV injection) or vehicle control (PBS) immediately following CLP-induced sepsis. At 24 h post-CLP, Heme-treated mice displayed significantly elevated endothelial senescence, reduced proliferation, increased apoptosis and decreased capillary density in the heart compared to vehicle-treated controls (Figs. 3D–F and S3A–F). In addition, echocardiographic analysis demonstrated that exogenous heme administration led to a significant reduction in cardiac function, as evidenced by decreased ejection fraction (EF) and fractional shortening (FS) (Fig. 3G–I). Western blot analysis further revealed significantly increased expression of senescence markers, including acetyl-p53, p21, and p16, in septic heart tissues treated with exogenous heme compared to control septic tissues (Fig. 3J, K). Collectively, these results suggest that elevated circulating heme not only correlates with but also exacerbates cardiac endothelial senescence and contributes to impaired cardiac function during sepsis.

### Heme exacerbates bacterial-induced endothelial senescence

**Fig. 4:**
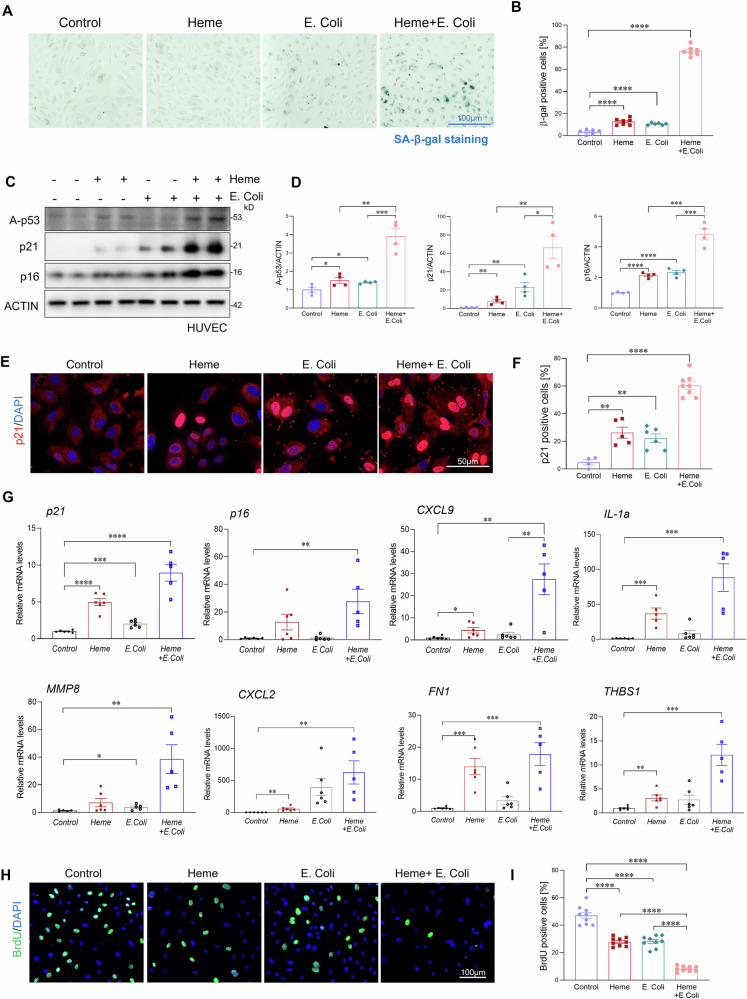
Heme exacerbates bacterial-induced endothelial senescence. **A**, **B** β-galactosidase staining and quantification of HUVECs treated with heme (10 µM), heat-killed *E. coli* (MOI:10), or both for 24 h (n = 6/group). Scale bars, 100 μm. **C**, **D** Western blot analysis and quantification of senescence markers p16, p21, and acetylated p53 in the indicated HUVEC groups (n = 4/group). **E**, **F** Immunofluorescence staining and quantification of p21 in the indicated HUVEC groups (n = 6/group). Scale bars, 50 μm. **G** qRT-PCR analysis of senescence-associated genes in the indicated HUVEC groups (n = 6/group). **H**, **I** BrdU incorporation assay and quantification in the indicated HUVEC groups (n = 6/group). Scale bars, 100 μm. All data are presented as mean ± SD. *P < 0.05, **P < 0.01, ***P < 0.001, ****P < 0.0001.

To determine whether heme directly contributes to endothelial senescence under septic conditions, and to mimic the in vivo environment of bacterial infection in the presence of elevated heme, we treated HUVECs with heme (10 µM), heat-killed bacteria (*E.coli*, MOI:10), or a combination of both for 6 and 24h. Notably, treatment with either heme or *E.coli* alone induced only mild endothelial senescence, as indicated by β-gal staining (Fig. 4A, B) and increased expression of senescence markers p16, p21, and acetyl-p53 (Fig. 4C, D). Specifically, heme treatment led to 12.6% senescence, while *E. coli* exposure resulted in 10.6% senescence in HUVECs. Strikingly, co-treatment with heme and *E. coli* resulted in a dramatic increase in cellular senescence, reaching 76.5% in HUVECs as shown by β-gal staining (Fig. 4A, B). Consistently, p21 immunostaining also revealed a robust increase in endothelial senescence in the co-treatment group compared with heme or *E.coli* alone (Fig. 4E, F). Additionally, the combination of heme and *E.coli* significantly upregulated the expression of senescence-associated secretory phenotype (SASP) factors, including CXCL9, IL-1α, MMP8, CXCL2, FN1, and THBS1 compared to either treatment alone(Fig. 4G). Effective vascular repair following injury is critical for cardiac functional recovery. To further assess whether endothelial senescence impairs this repair capacity, we evaluated endothelial cell migration, proliferation, and tube formation. Co-treatment with heme and E. coli significantly impaired endothelial cell function compared to single treatments or controls (Fig. 4H, I and S4A–D). These findings suggest that heme not only directly promotes endothelial senescence but also acts synergistically with bacterial components to amplify cellular senescence in endothelial cells, thereby exacerbating vascular dysfunction and impairing vascular repair capacity during sepsis. This severe cardiac endothelial senescence may also help explain why many septic patients experience long-term cardiomyopathy.

### Heme aggravates bacterial infection-induced STING activation

**Fig. 5:**
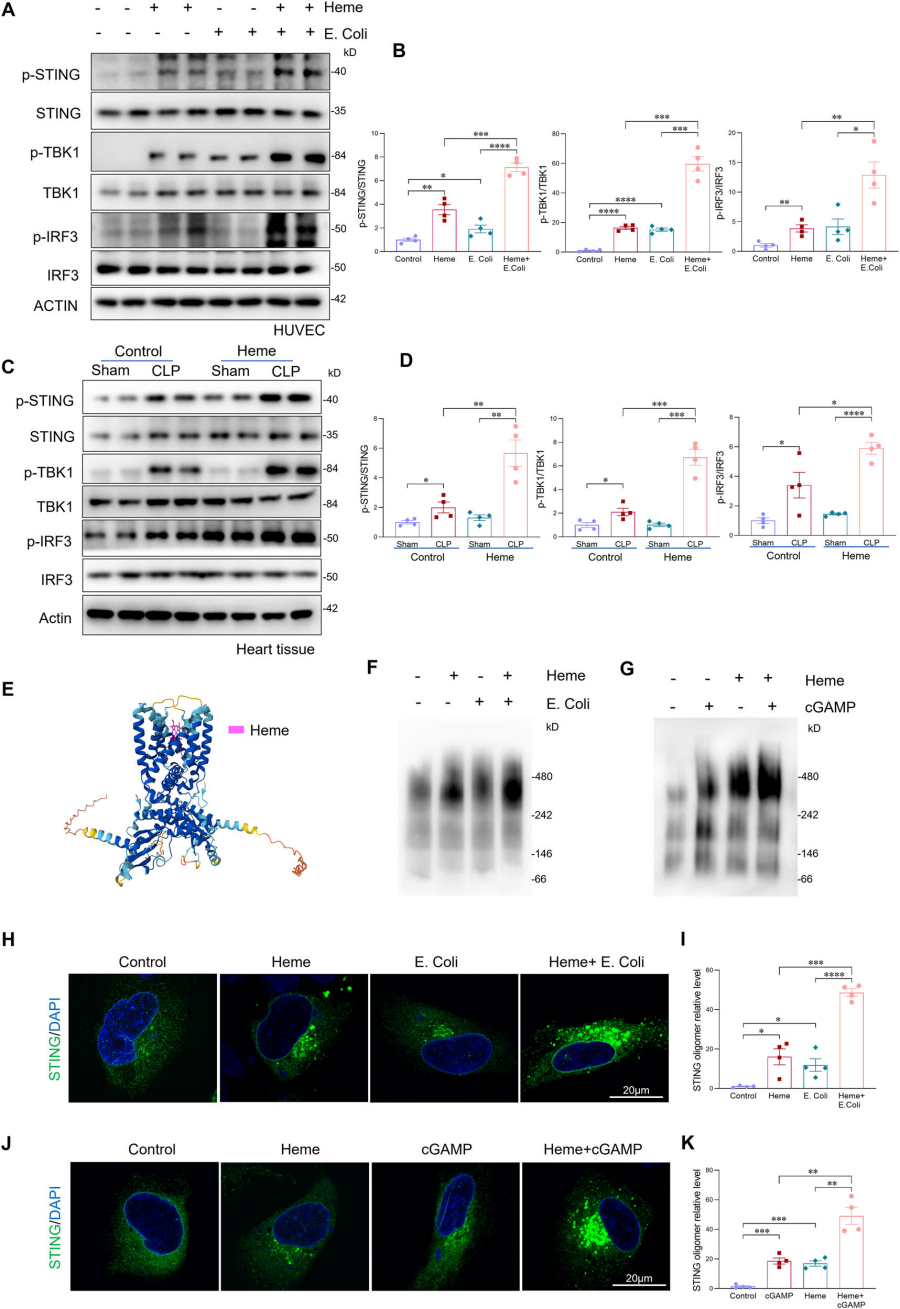
Heme aggravates bacterial infection-induced STING activation. **A**, **B** Western blot analysis and quantification of p-STING/STING, p-TBK1/TBK1, and p-IRF3/IRF3 in HUVECs treated with heme (10 µM), heat-killed *E. coli* (MOI:10), or both for 6 h (n = 6/group). **C**, **D** Western blot analysis and quantification of p-STING/STING, p-TBK1/TBK1, and p-IRF3/IRF3 in heart tissues from sham and septic mice treated with heme (n = 4/group). **E** Structural modeling of heme (pink) binding to the STING dimer complex using AlphaFold. **F**, **G** Native gel Western blot analysis of STING oligomerization in HUVECs treated with heme, *E. coli*, cGAMP, or their combinations. **H**–**K** Immunofluorescence staining and quantification of STING aggregates in HUVECs transfected with STING-HA and TBK1-Flag plasmids, followed by treatment with heme, *E. coli*, cGAMP, or their combinations. Scale bars, 20 μm. All data are presented as mean ± SD. *P < 0.05, **P < 0.01, ***P < 0.001, ****P < 0.0001.

To elucidate the mechanisms linking heme and bacterial-induced endothelial senescence, we examined the activation of the cGAS-STING pathway, a known driver of cellular senescence [22, 30, 32]. As shown in Fig. 5A, B, treatment of HUVECs with either heme or heat-killed *E. coli* alone resulted in only modest activation of the cGAS–STING pathway, as indicated by increased phosphorylation of STING, TBK1, and IRF3. However, co-treatment with heme and *E. coli* resulted in a marked enhancement of this pathway activation, with phosphorylation levels of STING, TBK1, and IRF3 increased by 2.7-, 2.9-, and 2.1-fold, respectively, compared with *E. coli* treatment alone (Fig. 5A, B). Consistently, in vivo studies revealed significantly enhanced activation of the cGAS–STING pathway in septic heart tissues following heme administration, compared to control septic heart tissues (Fig. 5C, D). To further determine the cell type in which STING is activated in the septic heart, we performed co-immunofluorescence staining for CD31 together with p-STING, p-TBK1, and p-IRF3. This analysis revealed a marked increase in p-STING, p-TBK1, and nuclear p-IRF3 signals predominantly within endothelial cells after sepsis, which was further enhanced by heme administration (Fig. S5A–C).

This robust activation of cGAS–STING signaling was not merely due to the additive effects of heme and *E. coli*, but rather suggested a potential synergistic mechanism. To investigate whether heme directly binds to STING and facilitates its oligomerization, a critical step required for downstream activation of the pathway, we generated a three-dimensional model of the STING dimer in complex with heme. As shown in Fig. 5E, heme was predicted to bind to the STING dimer. Based on this, we hypothesized that heme binding promotes STING activation by enhancing its oligomerization during bacterial infection. To test this, we assessed STING oligomerization using native gel western blot analysis following treatment with heme, *E. coli*, or both. In untreated cells, STING existed in a dimeric form. Treatment with either heme or *E. coli* individually induced low levels of STING oligomer formation. However, combined treatment with heme and *E. coli* resulted in a substantial increase in higher-order STING oligomers (Figs. 5F and S6A), indicating enhanced oligomerization and synergistic pathway activation. To further validate this mechanism, we treated HUVECs with the canonical STING ligand cGAMP in the presence or absence of heme. Consistent with our hypothesis, co-treatment with cGAMP and heme induced significantly greater STING oligomerization than either agent alone (Figs. 5G and S6B). In parallel, immunofluorescence staining revealed increased STING aggregation, particularly in cells co-treated with heme and *E. coli* or heme and cGAMP, supporting the formation of STING complexes (Figs. 5H–K and S6C, D). To determine whether heme-induced STING oligomerization enhances its interaction with TBK1, we transfected HUVECs with STING-HA and TBK1-Flag plasmids and assessed their interaction by co-immunoprecipitation. As shown in Fig. S6E–H, co-treatment with heme and *E. coli or* heme and cGAMP markedly enhanced the STING-TBK1 interaction compared with individual treatments. Collectively, these findings suggest that heme amplifies bacterial infection–induced activation of the cGAS-STING pathway by promoting STING oligomerization.

### STING activation contributes to sepsis-induced cardiac endothelial senescence

**Fig. 6:**
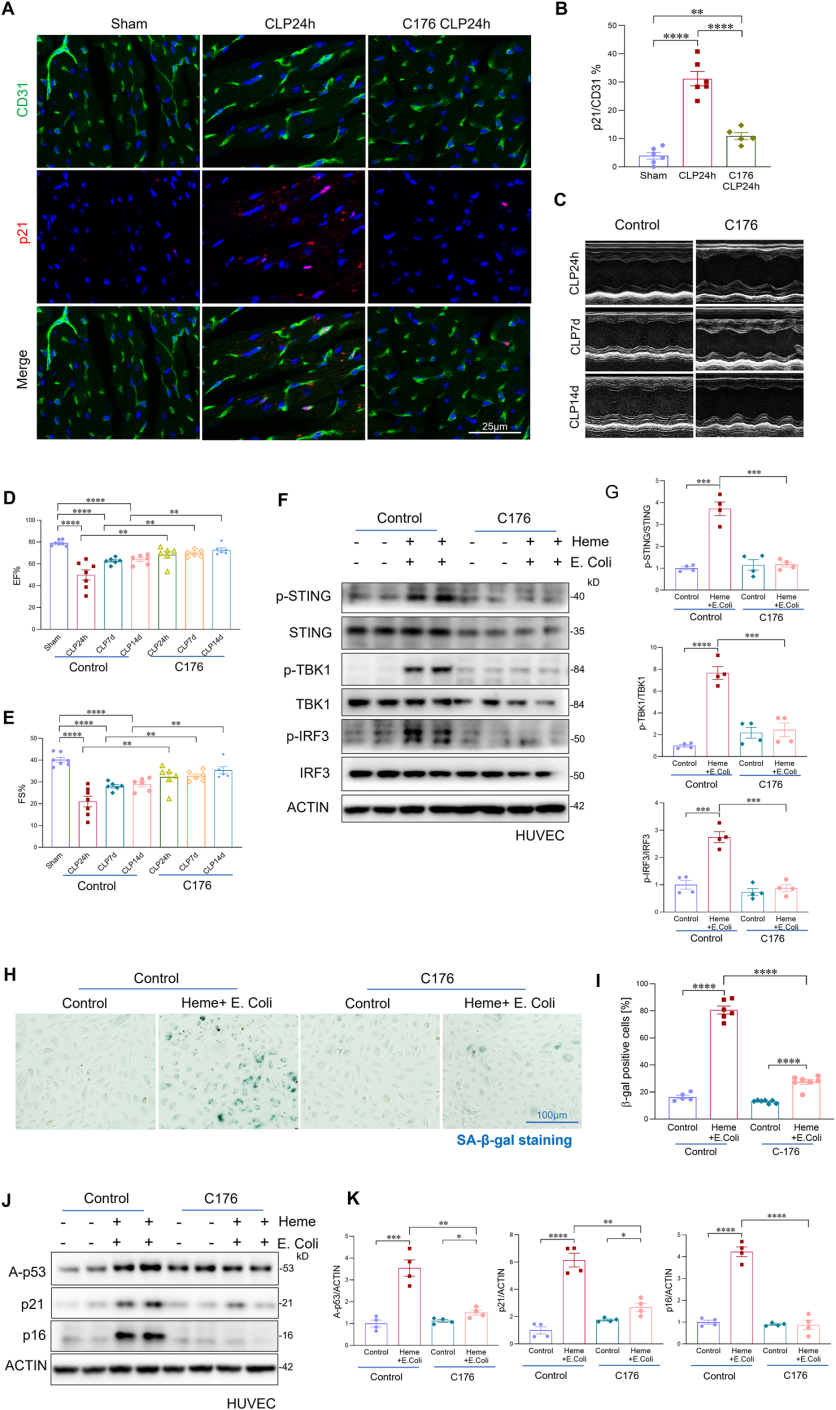
STING activation contributes to sepsis-induced cardiac endothelial senescence. **A**, **B** Co-immunofluorescence staining and quantification of the senescence marker p21 with the endothelial cell marker CD31 in cardiac tissues from sham and septic mice treated with a STING inhibitor or vehicle control at 24 h post-CLP (n = 6 mice/group). Scale bars, 25 μm. **C** Representative echocardiographic images assessing ejection fraction (EF) and fractional shortening (FS) at 24 h, 7 days, and 14 days post-CLP in mice treated with vehicle or STING inhibitor. **D**, **E** Echocardiographic assessment of EF and FS in septic mice treated with STING inhibitor or vehicle control at 24 h, 7 days, and 14 days post-CLP (n = 6 mice/group). **F**, **G** Western blot analysis and quantification of p-STING/STING, p-TBK1/TBK1, and p-IRF3/IRF3 in HUVECs treated with heme plus heat-killed *E. coli*, with or without the STING inhibitor C-176 (2.5 μM) (n = 4/group). **H**, **I** β-galactosidase (β-gal) staining and quantification of HUVECs treated with combined heme and *E. coli* with or without the STING inhibitor C-176 (n = 6/group). **J**, **K** Western blot analysis and quantification of senescence markers p16, p21, and acetylated p53 in the indicated HUVEC groups (n = 4/group). Scale bars, 100 μm. All data are presented as mean ± SD. *P < 0.05, **P < 0.01, ***P < 0.001, ****P < 0.0001.

We next investigated the role of cGAS-STING signaling in sepsis-induced cardiac endothelial senescence. Mice were treated with a STING inhibitor (C-176) or vehicle control immediately following CLP-induced sepsis. Heart tissues were collected 24 h post-CLP. Co-staining of phosphorylated STING, TBK1, and IRF3 with CD31 demonstrated that C-176 effectively inhibited sepsis-induced STING signaling activation in cardiac endothelial cells (Fig. S8A–C). As shown in Fig. 6A, B, STING inhibition significantly reduced endothelial senescence in cardiac tissue, as evidenced by decreased p21 and p16 expression compared with vehicle-treated controls (Figs. 6A, B and S7A, B). Moreover, echocardiographic analysis revealed that pharmacological inhibition of STING markedly improved cardiac function in septic mice, as indicated by increased ejection fraction (EF) and fractional shortening (FS) (Fig. 6C–E). To further determine whether long-term STING inhibition promotes sustained cardiac recovery, we treated septic mice with C-176 or vehicle every other day for 2 weeks. Long-term STING inhibition significantly reduced cardiac endothelial senescence and fibrotic remodeling, and improved cardiac functional recovery (Figs. 6C–E and S7C–F, S10A). To assess whether STING activation directly contributes to heme and bacterial-induced endothelial senescence, we treated HUVECs with heme combined with *E. coli*, or with septic plasma in the presence or absence of a STING inhibitor. STING inhibition significantly suppressed activation of the cGAS–STING pathway in response to heme–bacterial challenge, as shown by reduced phosphorylation of STING, TBK1, and IRF3 (Fig. 6F, G*)*. In parallel, STING inhibition markedly attenuated endothelial senescence induced by either heme plus *E.coli* or septic plasma, as indicated by reduced β-galactosidase activity (Figs. 6H, I and S9A, B). Consistent with these findings, STING inhibition reduced immunostaining of p21 (Fig. S9C, D) and lowered protein expression of senescence markers p21, p16, and acetylated p53 (Fig. 6J, K). Additionally, STING inhibition mitigated the detrimental effects of heme and *E. coli* on endothelial reparative function, significantly restoring endothelial proliferation and tube formation capacity (Fig. S9E–H). Collectively, these findings demonstrate that cGAS-STING signaling plays a critical role in driving endothelial senescence and cardiac dysfunction during sepsis. Inhibition of STING enhances endothelial repair capacity and promotes cardiac functional recovery, underscoring its therapeutic potential in sepsis-associated cardiovascular complications.

### Hemopexin upregulation alleviates sepsis-induced endothelial senescence and cardiac dysfunction

**Fig. 7:**
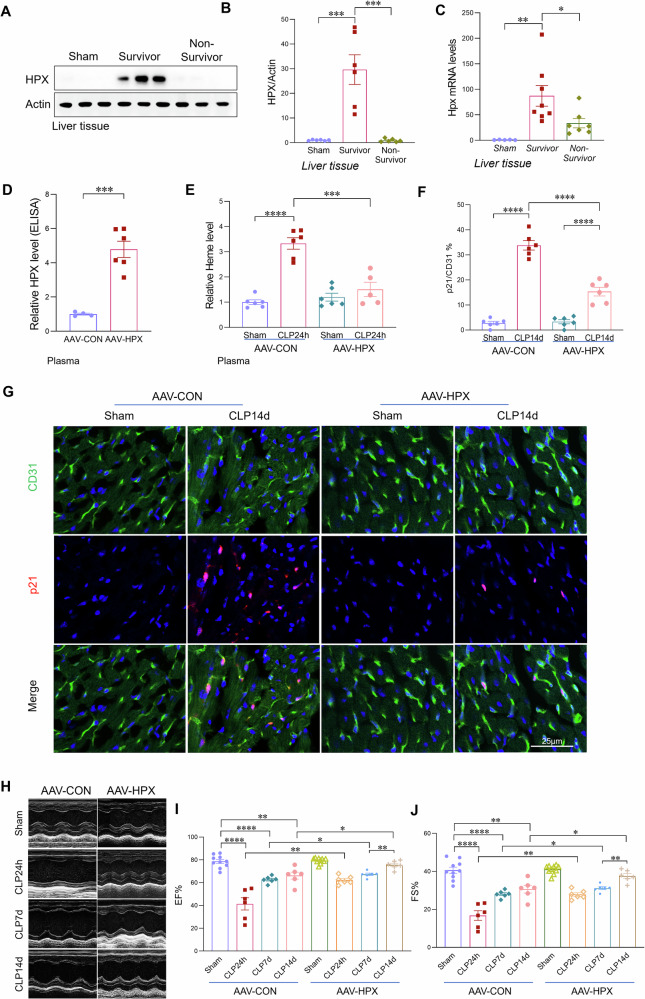
Hemopexin upregulation alleviates sepsis-induced endothelial senescence and cardiac dysfunction. **A**, **B** Western blot analysis and quantification of HPX expression in liver tissues from sham mice, septic survivors, and septic non-survivors at 24 h post-CLP (n = 6/group). **C** qRT-PCR analysis of HPX expression in liver tissues from sham mice, septic survivors, and septic non-survivors (n = 6–8/group). **D** ELISA confirmation of HPX overexpression in plasma following AAV–HPX administration (1 × 10¹¹ GC/mouse), with AAV-CON as the control (n = 6/group). **E** Plasma free heme levels measured 24 h post-CLP in AAV-HPX and AAV-CON mice (n = 6–8/group). **F**, **G** Co-immunofluorescence staining and quantification of the senescence marker p21 with the endothelial cell marker CD31 in heart tissues from the indicated groups. Scale bars, 25 μm. **H**–**J** Echocardiographic assessment of ejection fraction (EF) and fractional shortening (FS) in the indicated groups at 24 h, 7 days, and 14 days post-sepsis (n = 6–8/group). All data are presented as mean ± SD. *P < 0.05, **P < 0.01, ***P < 0.001, ****P < 0.0001.

Hemopexin (HPX), a liver-derived heme scavenger, plays a critical role in neutralizing excess free heme and preventing its cytotoxic effects [22, 23]. Given our earlier findings that circulating free heme levels were markedly elevated in non-survivors compared with survivors, we next examined HPX expression across these groups. Notably, HPX levels were significantly higher in survivors than in non-survivors (Fig. 7A–C), suggesting a protective role of HPX during sepsis. To determine whether enhancing HPX expression could attenuate sepsis-induced endothelial senescence and cardiac dysfunction, we administered AAV-HPX (1 × 10¹¹ GC/mouse) to overexpress HPX, with AAV-CON as a control. Two weeks after AAV delivery, the mice were subjected to CLP sepsis. An ELISA assay confirmed successfully elevated HPX levels in plasma (Fig. 7D). Following sepsis induction, mice treated with AAV–HPX exhibited significantly reduced circulating free heme levels (Fig. 7E). Increased HPX expression attenuated cardiac endothelial senescence, as evidenced by decreased expression of p21 and p16 at both 24 h and 14 days post-sepsis (Figs. 7F, G and S11A–D, S12A, B). Consistently, Ki67 staining showed that endothelial cell proliferative capacity was enhanced in septic mice with increased HPX expression, compared to control septic mice (Fig. S12C, D). Furthermore, AAV-HPX treatment improved cardiac functional recovery, as indicated by increased ejection fraction (EF) and fractional shortening (FS) on echocardiographic analysis at 24 h, 7 days, and 14 days following sepsis (Fig. 7H–J). In addition, HPX overexpression also protected against cardiac fibrotic remodeling at day 14 post-CLP (Fig. S13C). Collectively, these findings demonstrate that enhancing heme clearance through increased HPX expression mitigates sepsis-induced cardiac endothelial senescence and promotes cardiac functional recovery.

## Discussion

**Fig. 8:**
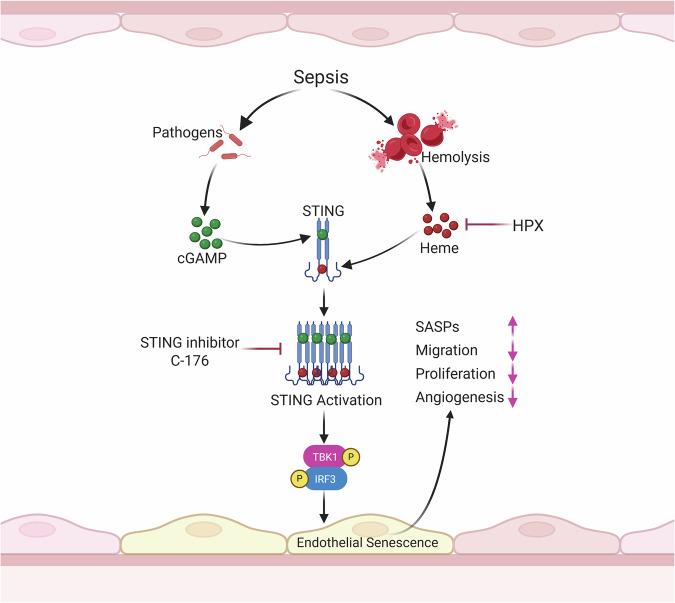
Schematic illustration. Heme drives cardiac endothelial senescence in sepsis through STING activation. During sepsis, free heme—a byproduct of hemolysis—acts as a novel ligand for STING, amplifying bacterial infection–induced STING polymerization and downstream signaling activation, thereby promoting endothelial senescence. Senescent endothelial cells contribute to sepsis-induced cardiac dysfunction by upregulating senescence-associated secretory phenotype (SASP) factors and impairing vascular repair through diminished endothelial proliferation, migration, and angiogenesis. Pharmacological inhibition of STING or hemopexin (HPX)-mediated heme clearance attenuates endothelial senescence and improves cardiac functional recovery under septic conditions.

Sepsis-induced cardiac dysfunction is a life-threatening complication that substantially contributes to sepsis-related mortality [3–5]. Despite its clinical significance, the underlying cellular and molecular mechanisms remain incompletely understood. In this study, we identify cardiac endothelial senescence as a key pathological feature of sepsis and demonstrate that elevated levels of free heme, a byproduct of hemolysis, play a pivotal role in driving this response. Our findings reveal a novel role of heme in activating the cGAS-STING pathway and promoting endothelial senescence, thereby impairing vascular repair and contributing to cardiac dysfunction during sepsis (Fig. 8). These results highlight both STING inhibition and heme clearance as promising therapeutic strategies for preserving cardiovascular function in septic patients.

Endothelial senescence, a hallmark of aging, contributes to the pathogenesis of several cardiovascular diseases (CVDs), including stroke, atherosclerosis, and hypertension [15–17, 19–21, 38]. However, whether endothelial senescence occurs in sepsis and contributes to sepsis-associated cardiac dysfunction has not been previously explored. In this study, we show for the first time that cardiac senescence is markedly elevated during sepsis, with endothelial cells as the predominant senescent population. Notably, the extent of endothelial senescence correlated with disease severity and was significantly greater in non-survivors. Senescent endothelial cells exhibited impaired proliferative, angiogenic, and migratory capacities, compromising vascular repair and cardiac recovery after sepsis. These findings may explain the heightened long-term cardiovascular risk in sepsis survivors. A major advance of this study is the identification of free heme as a key driver of cardiac endothelial senescence. We demonstrate that circulating free heme levels correlate with the severity of endothelial senescence and cardiac dysfunction in septic mice. Moreover, administration of exogenous heme exacerbated both senescence and cardiac impairment, confirming its causative role in this process.

A key strength of this study is the discovery that heme amplifies bacterial-induced cGAS–STING activation, a known driver of cellular senescence [22, 30, 32, 39]. Co-treatment with heme and bacterial components synergistically enhanced cGAS–STING signaling. A previous study showed that compound C53 binds a cryptic pocket within the STING transmembrane domain, located between the dimer subunits [40]. In the presence of cGAMP, this interaction induces robust STING oligomerization and activation [40]. Using AlphaFold protein–ligand binding prediction, we found that heme also binds within the cryptic pocket of STING, suggesting that it could promote STING activation by facilitating oligomerization, similar to C53. As anticipated, heme significantly enhanced STING oligomerization and activation during bacterial challenge. This effect was further validated by co-treatment with cGAMP, confirming that heme augments STING signaling through oligomerization. These results provide compelling evidence that pathogen- and damage-associated signals orchestrate a maladaptive endothelial senescence response during sepsis.

Hemopexin (HPX), a liver-derived heme-binding protein, functions as a scavenger of free heme [22, 23, 25]. Septic patients with lower hemopexin levels show increased mortality compared with those with higher levels. In our study, we found that HPX levels were significantly higher in survivors than in non-survivors. Notably, increasing HPX expression significantly reduced circulating free heme levels, attenuated cardiac endothelial senescence, decreased fibrotic remodeling, and facilitated cardiac functional recovery in septic mice. These findings support the concept that enhancing heme clearance represents a viable strategy to mitigate organ dysfunction in sepsis.

Overall, our study has several important implications. First, it highlights cardiac endothelial senescence as a pathological hallmark and therapeutic target in septic cardiomyopathy. Second, it identifies free heme as a pathogenic factor that integrates hemolysis and immune activation to exacerbate endothelial damage and cardiac injury. Finally, it supports the therapeutic potential of targeting the cGAS–STING pathway or enhancing heme clearance to reduce vascular dysfunction and improve cardiac functional recovery.

There are limitations to consider. First, although we focused on cardiac endothelial senescence, senescence in other organs such as the lungs and kidneys was not examined and may also contribute to systemic dysfunction during sepsis. Second, the predicted interaction between heme and STING was based on computational modeling using AlphaFold. Further structural validation is required to confirm this binding and its functional consequences. Third, the use of systemic STING inhibitor may impair host defense against pathogens. More selective approaches, such as endothelial targeted delivery, may help preserve antimicrobial immunity while limiting endothelial senescence, these approaches are worth further investigation.

In conclusion, our study identifies a novel heme–STING–senescence axis that drives cardiac endothelial dysfunction during sepsis. By linking hemolysis to vascular injury and cardiac impairment, these findings provide a compelling rationale for therapeutically targeting heme clearance and STING signaling to preserve cardiovascular integrity and improve outcomes in sepsis.

## Materials and methods

Detailed descriptions of materials, methods, animal studies, and statistical analysis are provided in the Supplementary Materials.

## Supplementary information

Supplemental data

Original Data

## Data Availability

All data are available in the main text or the supplementary materials. All original data generated or analyzed during this study are available from the corresponding author upon request.
