# Supplementary material for: Heme drives cardiac endothelial senescence in sepsis via STING activation
Source: Cell Death Dis. 2025 Dec 18;17(1):108. doi: 10.1038/s41419-025-08370-w (PMC12847929; doi:10.1038/s41419-025-08370-w)

Figure. 1

D

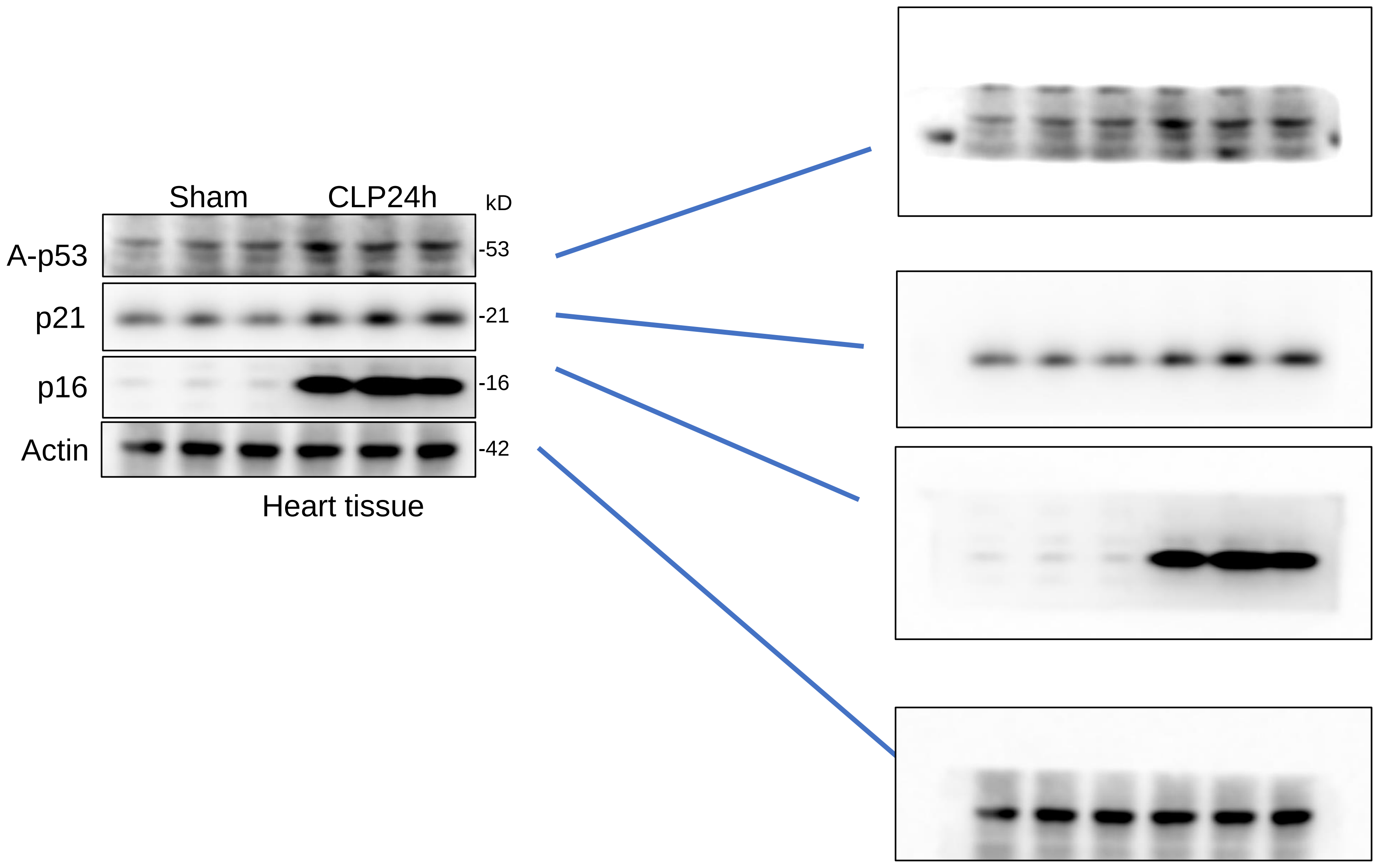

F

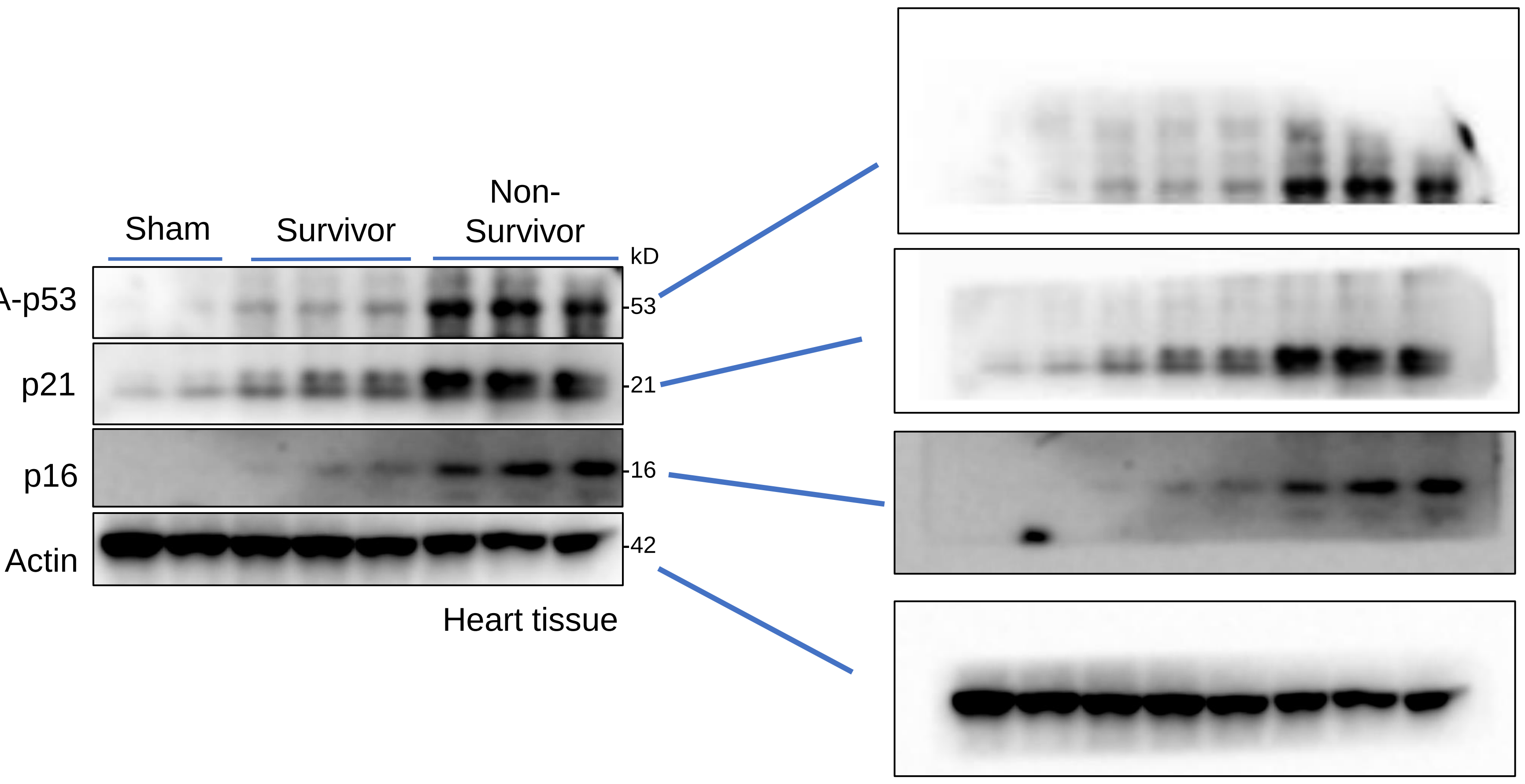

Figure. 3

I

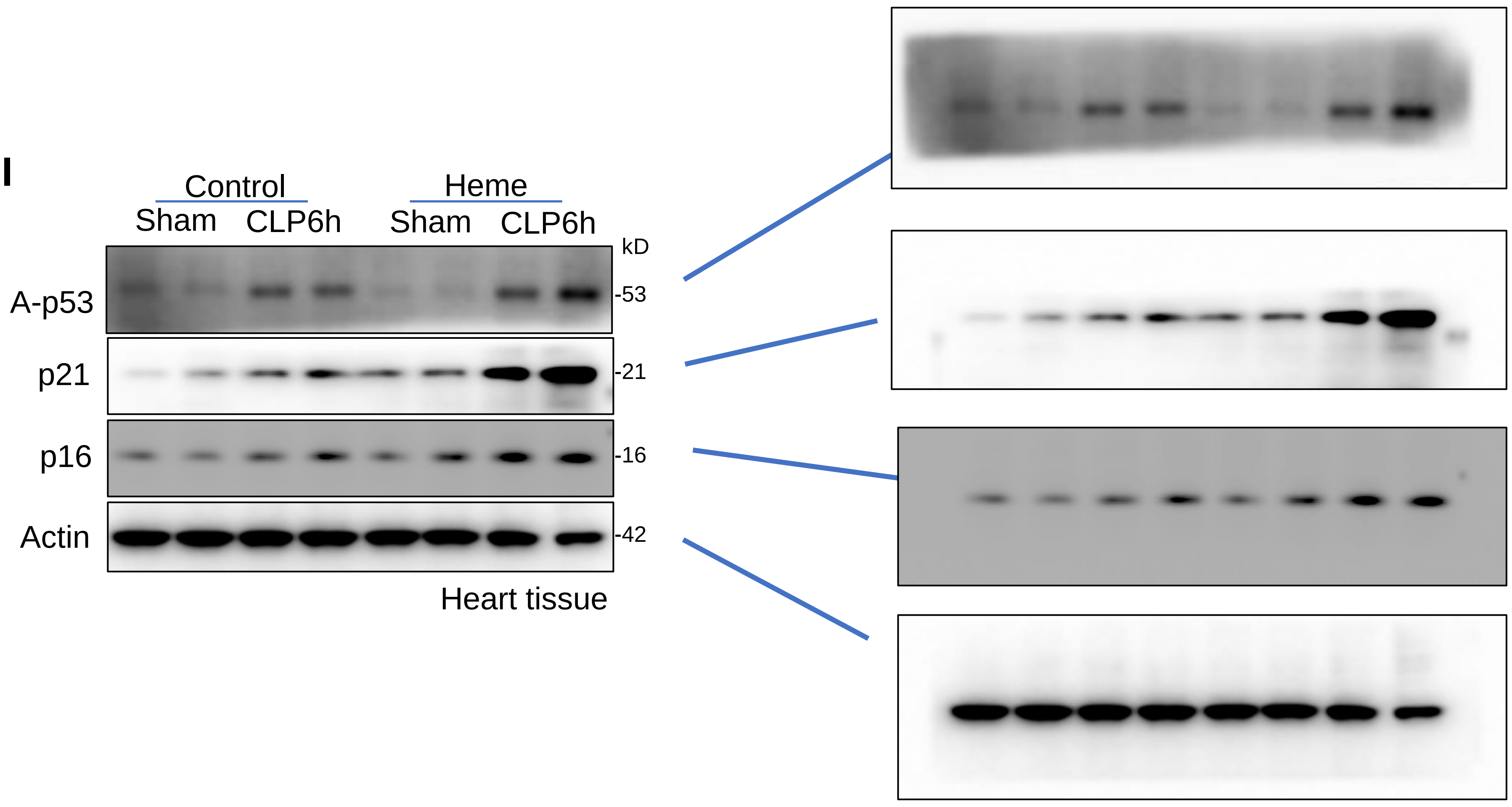

Figure. 4

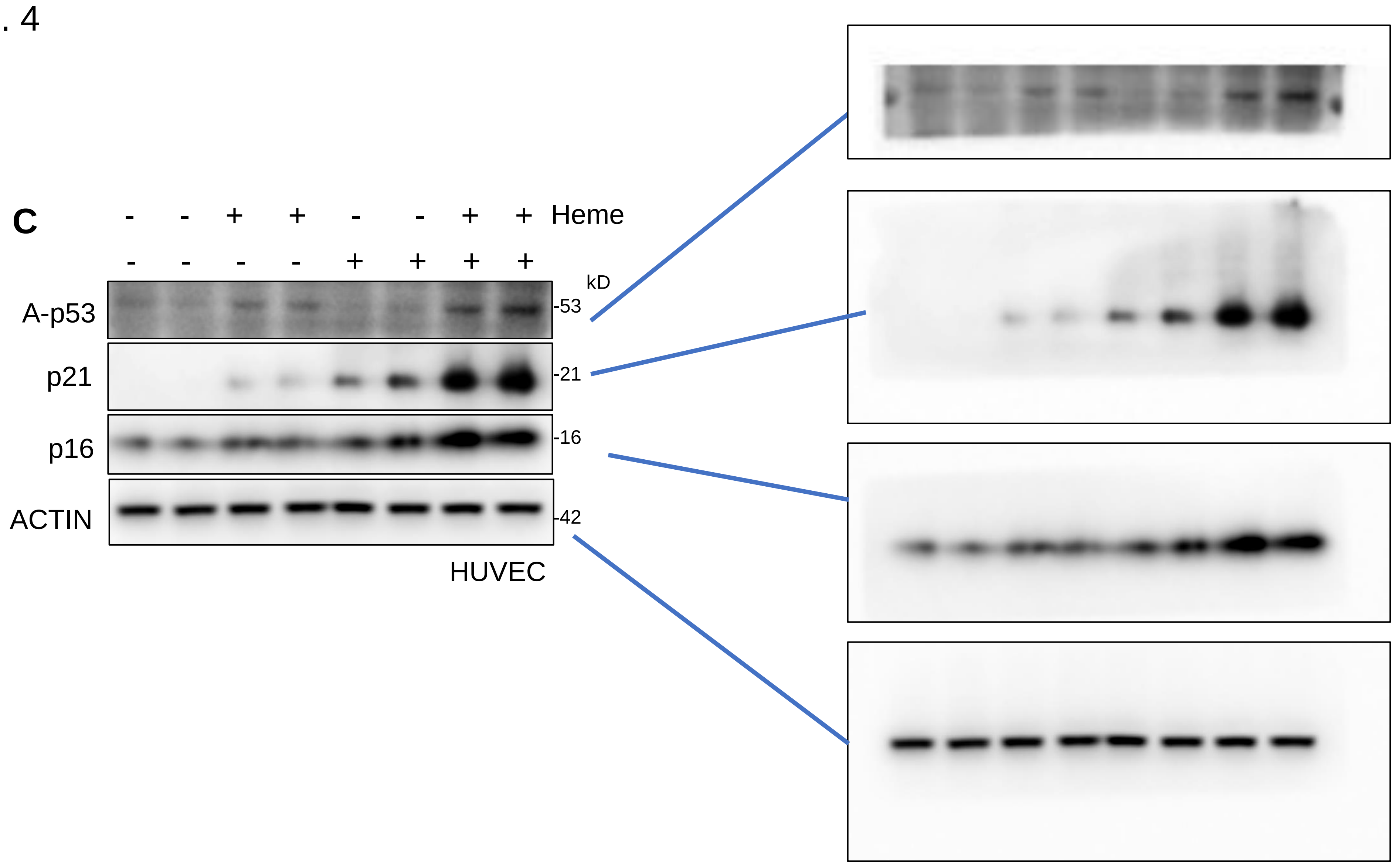

Figure. 5

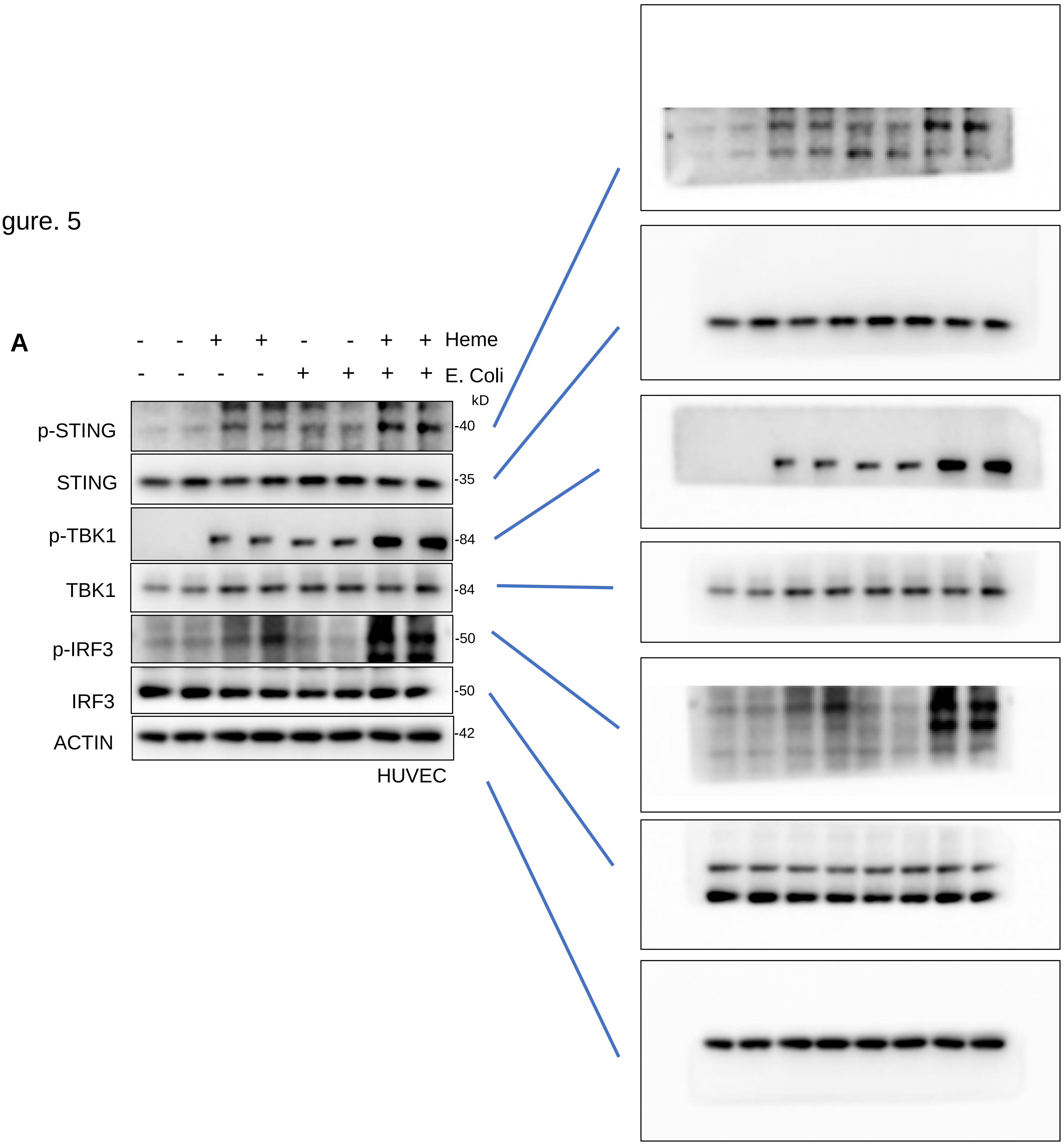

Figure. 5

D

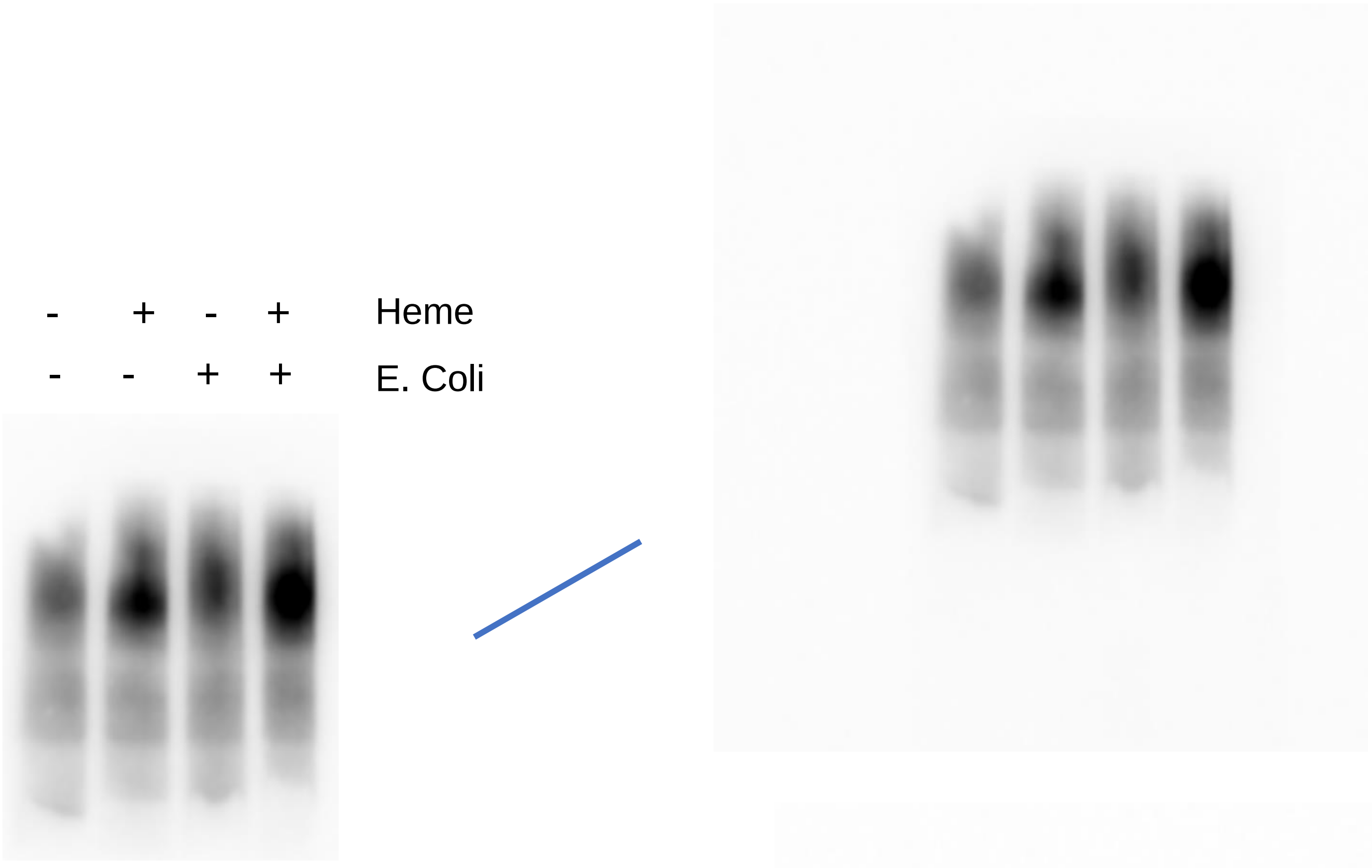

- + - + Heme
- - + + E. Coli

E

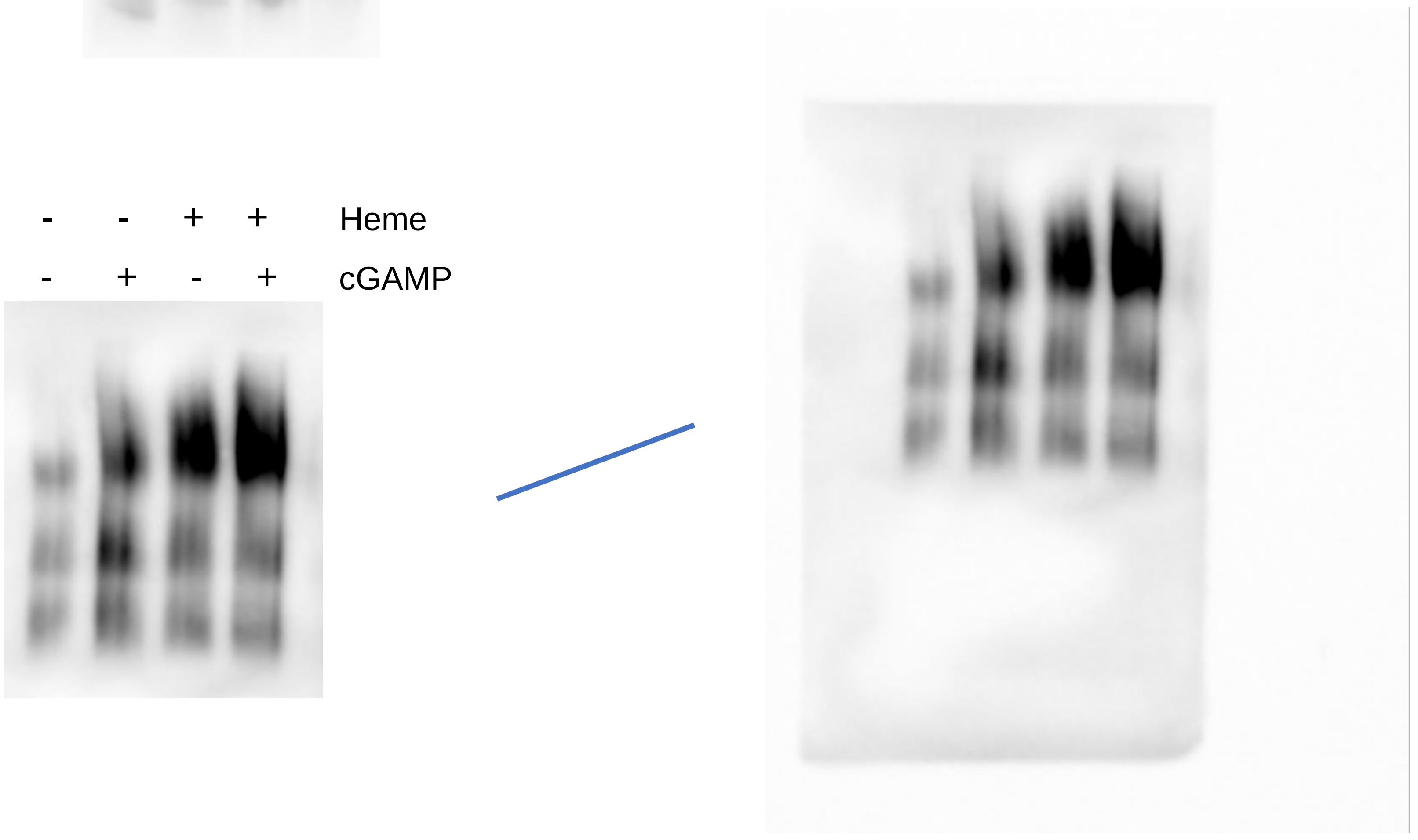

- - + + Heme
- + - + cGAMP

H

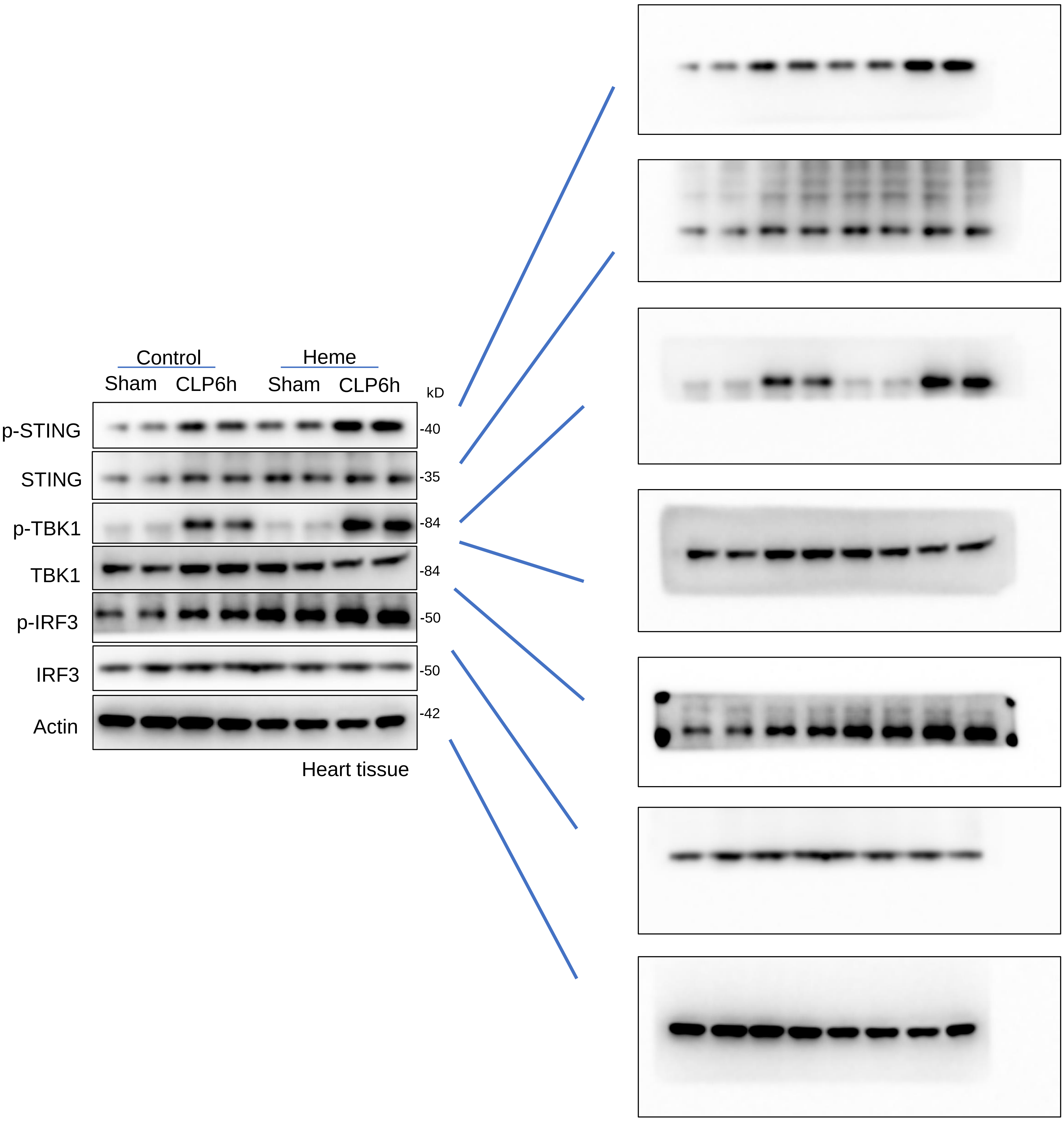

Control
Heme
Sham CLP6h Sham CLP6h
kD
p-STING -40
STING -35
p-TBK1 -84
TBK1 -84
p-IRF3 -50
IRF3 -50
Actin -42
Heart tissue

Figure. 6

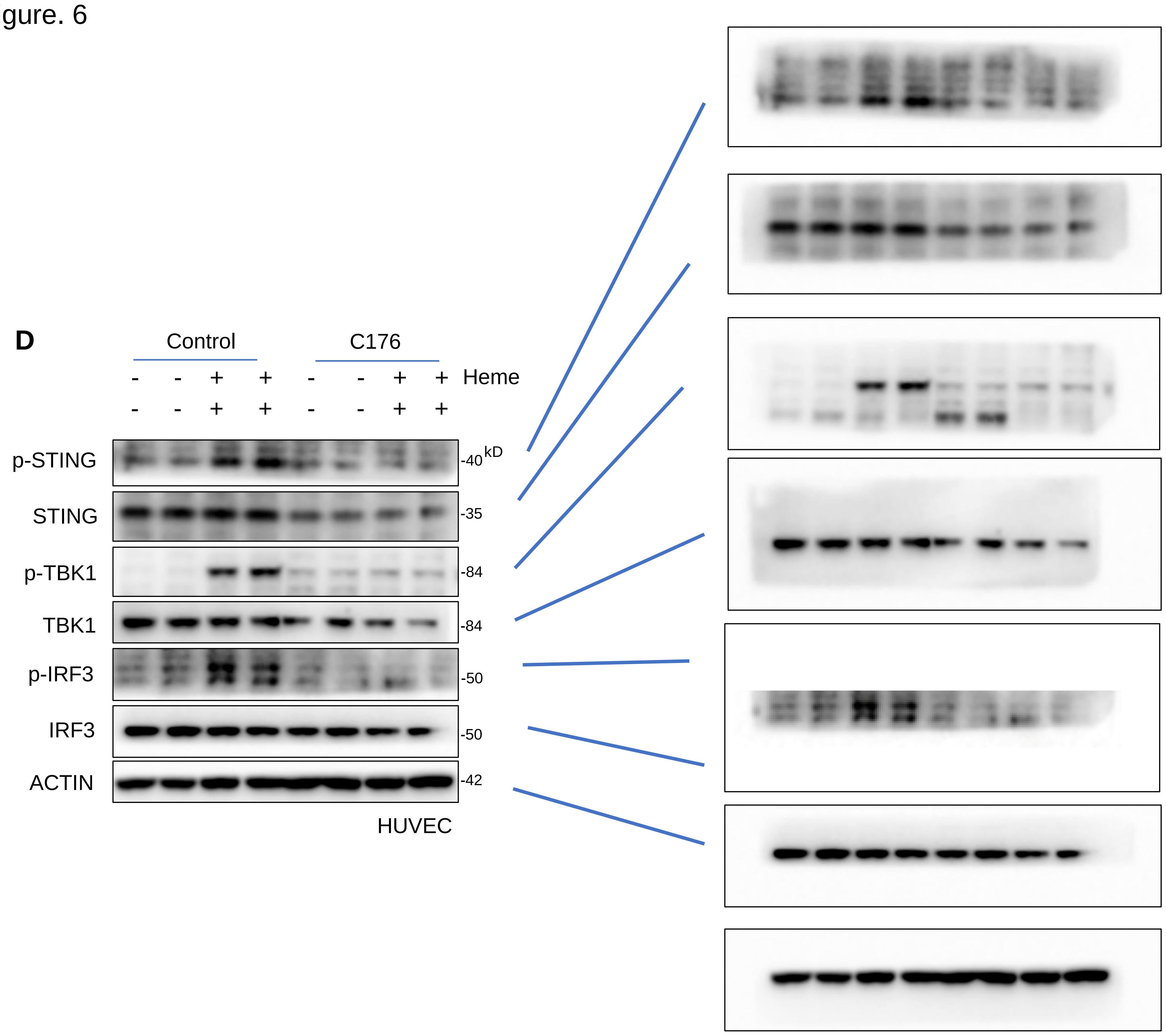

D
Control
C176
- - + + - - + + Heme
- - + + - - + +
p-STING
STING
p-TBK1
TBK1
p-IRF3
IRF3
ACTIN
kD
-40
-35
-84
-84
-50
-50
-42
HUVEC

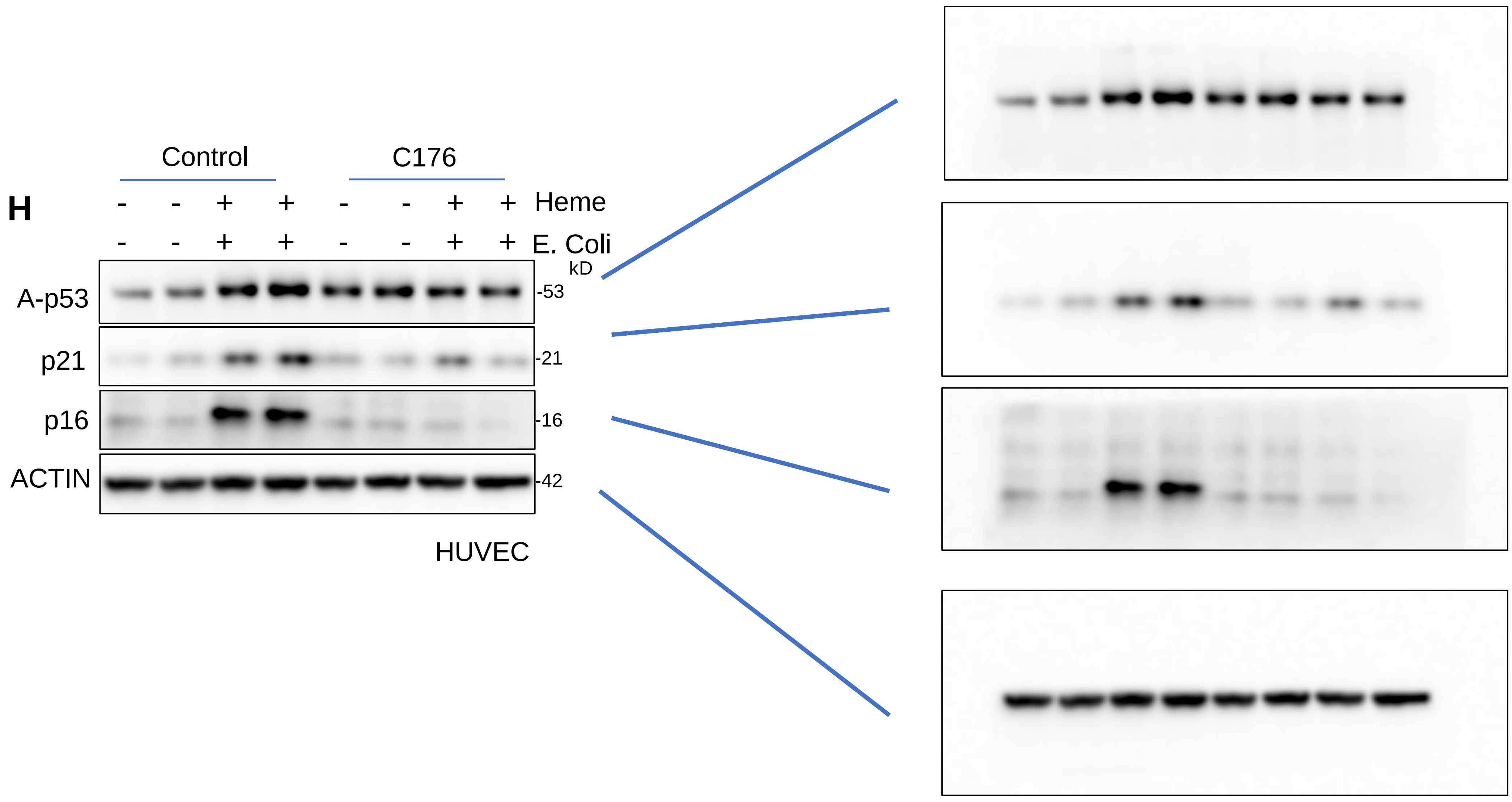

H
Control
C176
- - + + - - + + Heme
- - + + - - + + E. Coli
kD
A-p53
p21
p16
ACTIN
-53
-21
-16
-42
HUVEC

Figure. 7

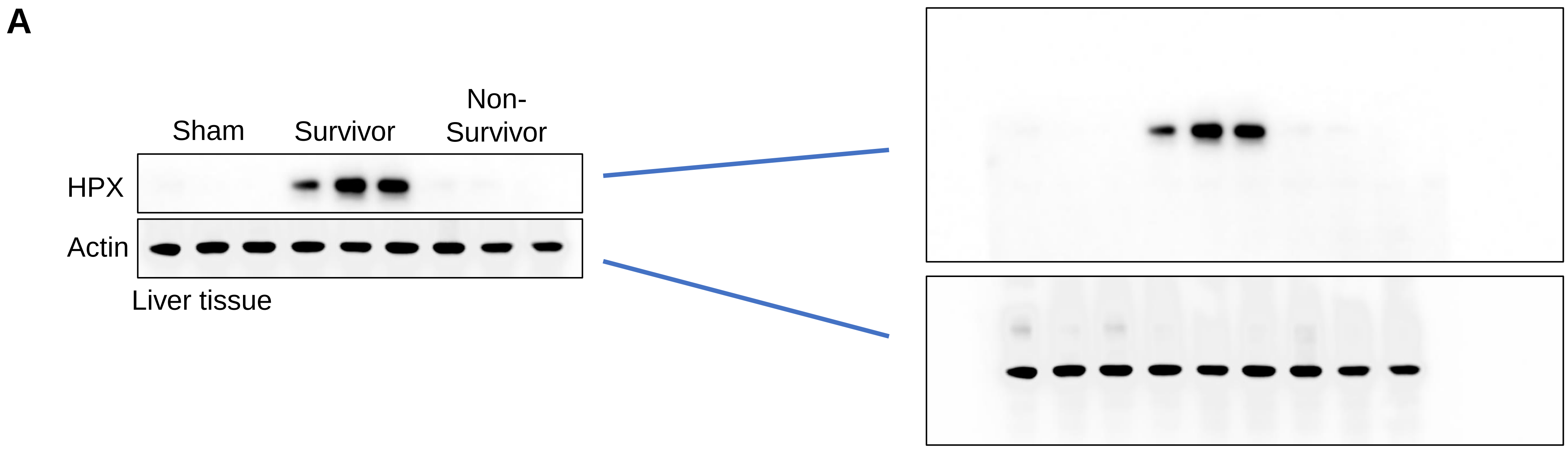

Figure. S4

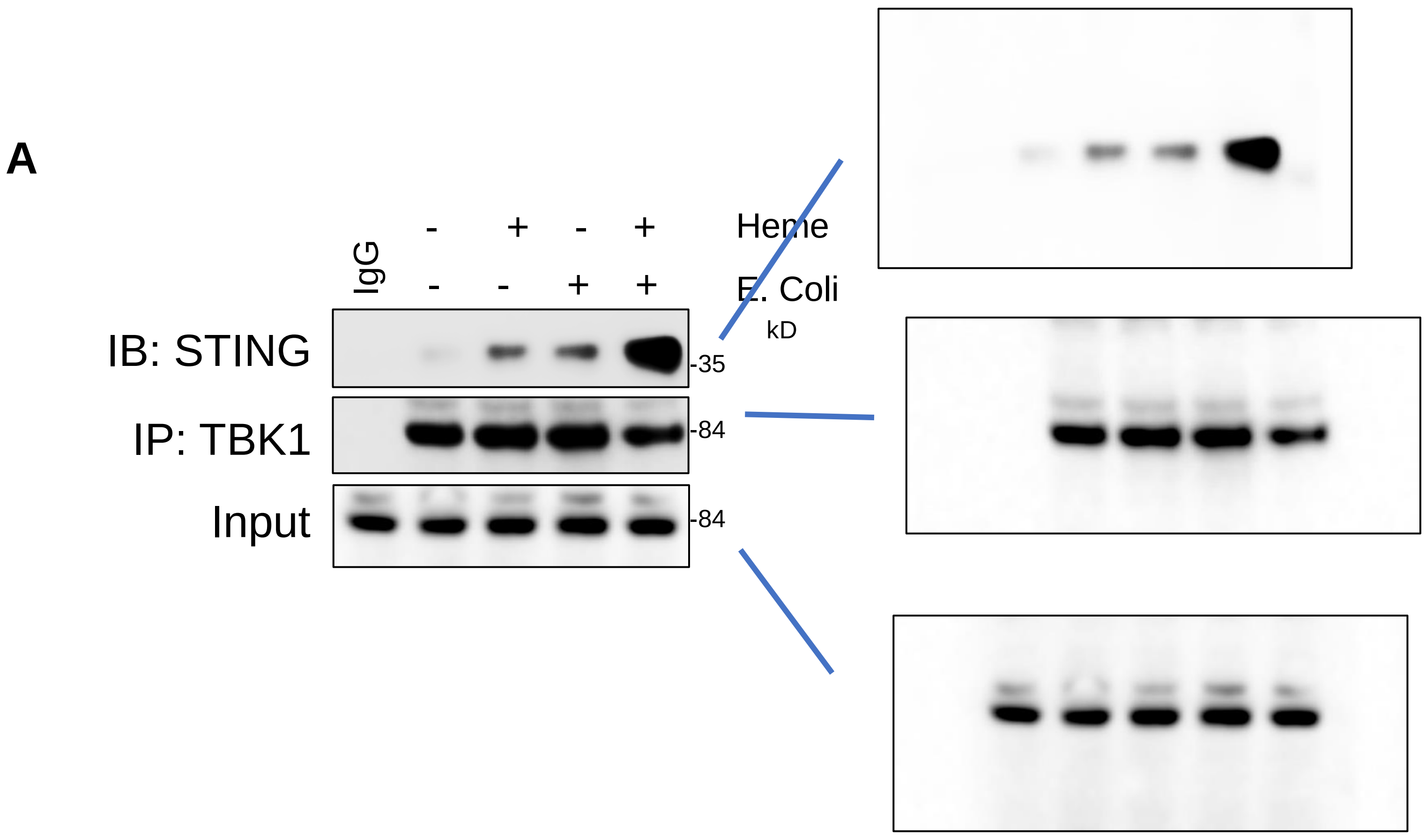

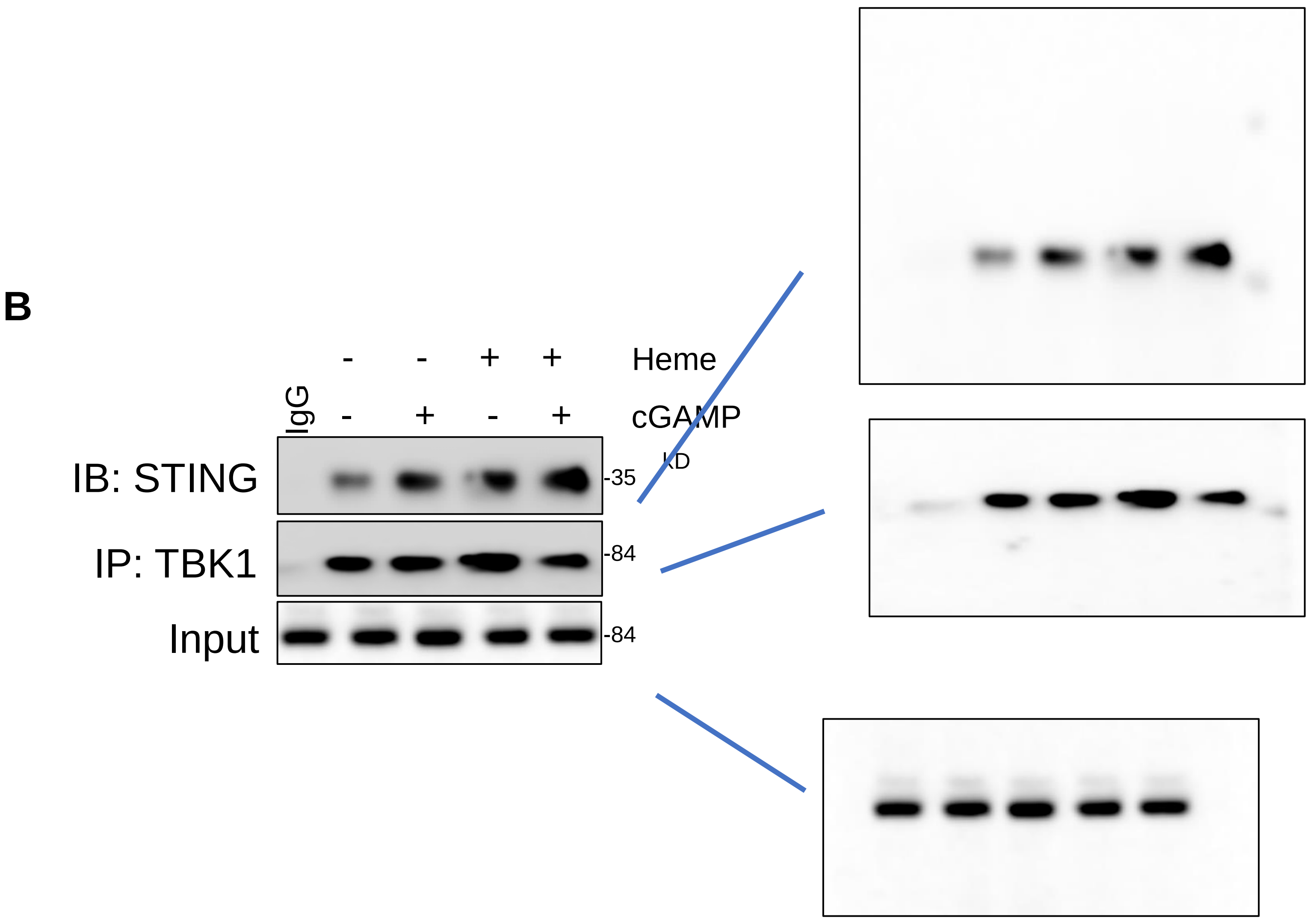

Figure. S4

C

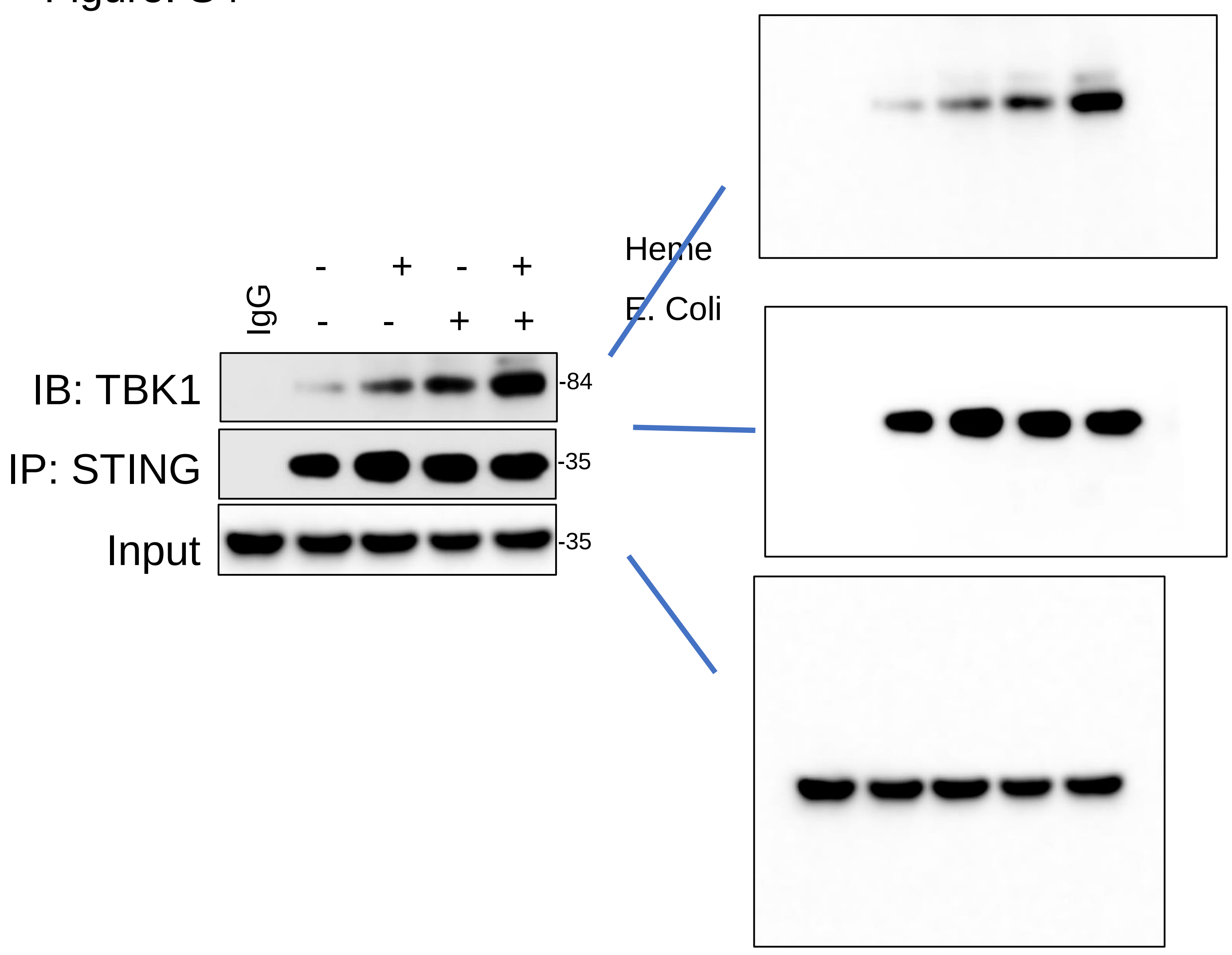

D

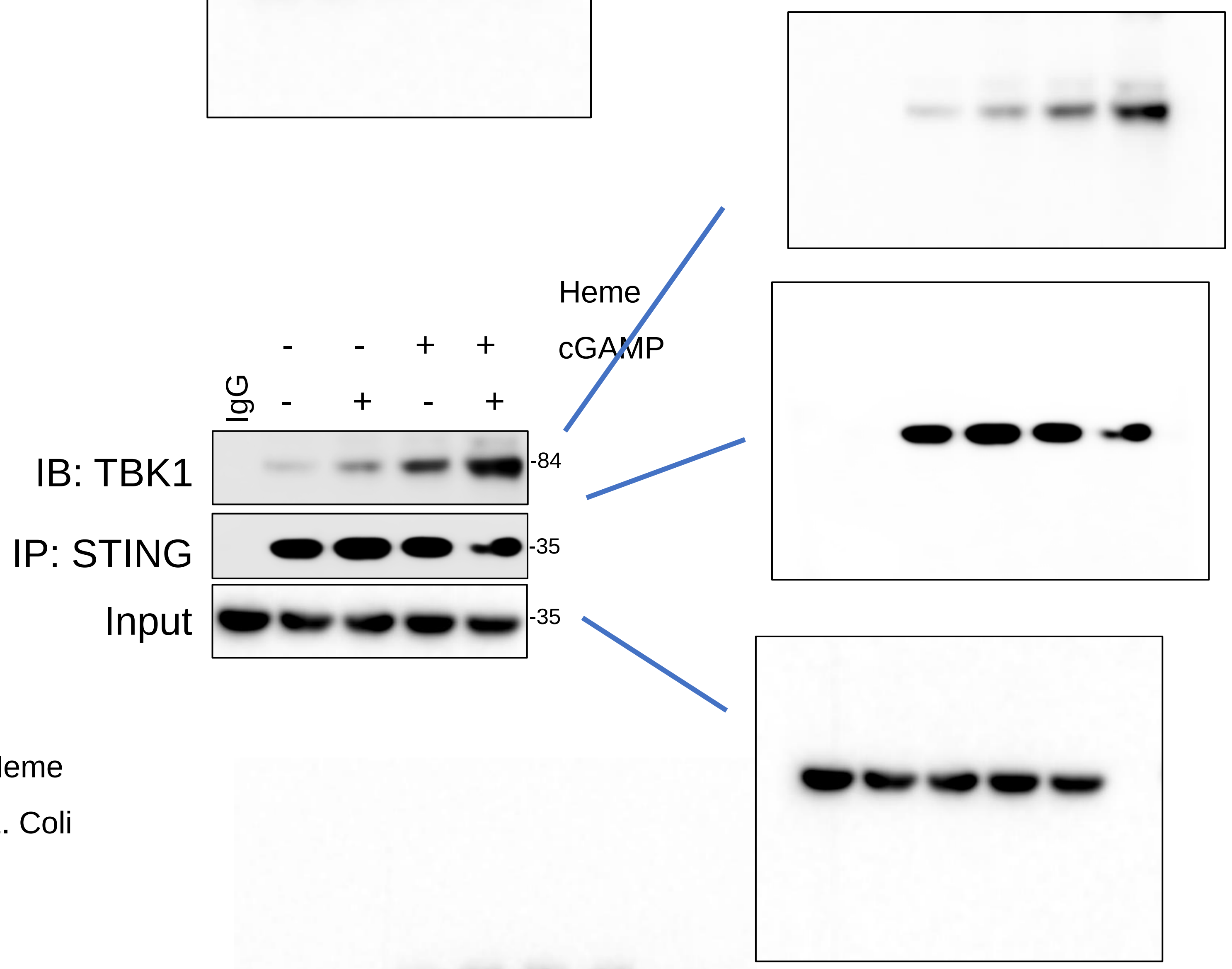

E

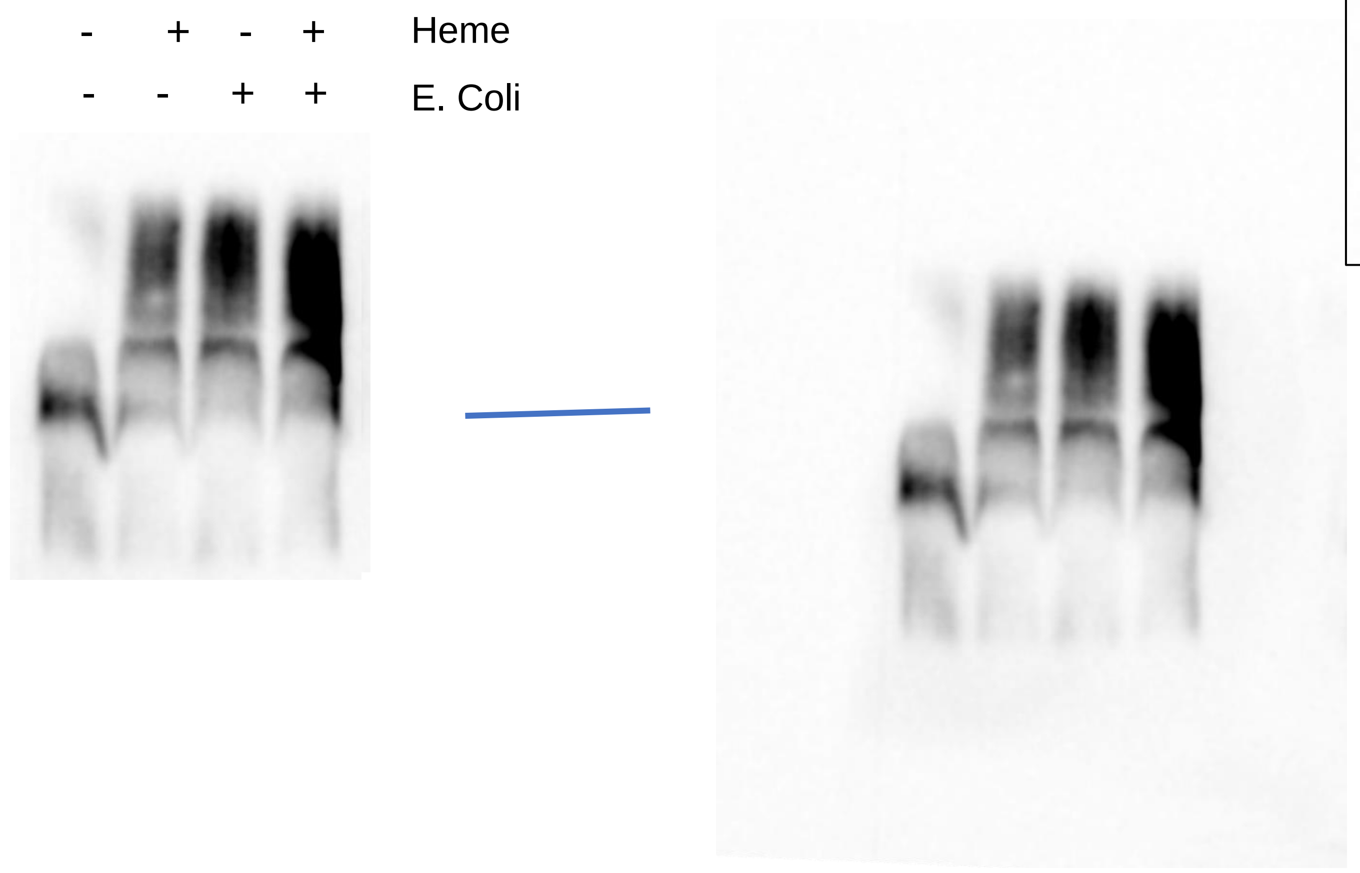

F

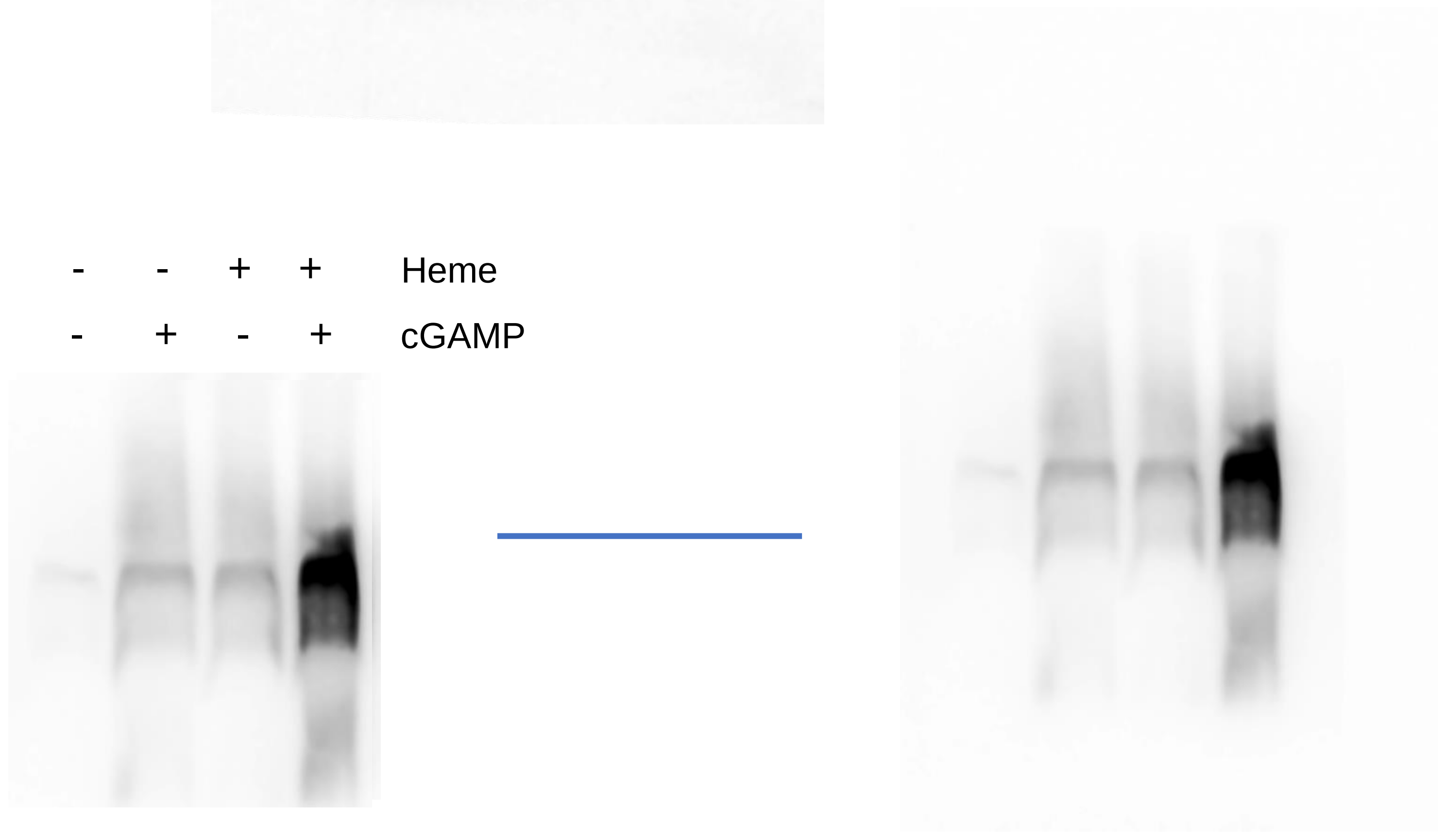

Supplement: Supplementary file 2 — Original Data [file 41419_2025_8370_MOESM2_ESM.pdf]
