# Supplementary material for: Heme drives cardiac endothelial senescence in sepsis via STING activation
Source: Cell Death Dis. 2025 Dec 18;17(1):108. doi: 10.1038/s41419-025-08370-w (PMC12847929; doi:10.1038/s41419-025-08370-w)
Supplement: Supplementary file 1 — Supplemental data [file 41419_2025_8370_MOESM1_ESM.docx]

**Heme drives cardiac endothelial senescence in sepsis via STING activation**

Tingting Li, Peilin Zhu, Jialing Wang, Tao Zhang, Joseph Adams, Fei Tu, Chloe Garbe, Xiaojin Zhang, Li Liu, Krishna Singh, David L Williams, Chuanfu Li, Xiaohui Wang

**Table of contents**

Supplementary methods and Materials.....................................................................................2

Supplementary figures...............................................................................................................9

**Animals and Housing**

Wild-type C57BL/6 mice were obtained from Jackson Laboratory and housed at the Institutional Animal Care and Use Facility at East Tennessee State University. Male and female mice aged 3-5 months were used in this study. All animal care and experimental procedures were approved by the ETSU Committee on Animal Care and Use and adhered to NIH guidelines to ensure humane treatment.

**Sepsis Model: Cecal Ligation and Puncture (CLP)**

Polymicrobial sepsis was induced using the cecal ligation and puncture (CLP) model as described previously. Briefly, mice were anesthetized with isoflurane and positioned on a heated surgical pad. Following a midline incision, approximately one-third of the cecum was ligated and punctured once using a 23-gauge needle. After repositioning the cecum, the abdominal cavity was closed. The peritoneal wall was sutured with sterile 4-0 Dafilon sutures and the skin was closed with a surgical staple (Autoclip 9 mm; Cat:12020-09). Sham-operated mice underwent similar procedures without ligation or puncture. Following CLP, mice were administered a single subcutaneous dose of resuscitative fluid. Liver tissues and plasma were collected at 6 and 24 hours post CLP for immunostaining, heme level quantification, and bacterial colony analysis.

**Cell culture and treatment**

Human umbilical vein endothelial cells (HUVECs) were obtained from the American Type Culture Collection (ATCC, PCS-100-013) and cultured in Vascular Cell Basal Medium (ATCC, PCS-100-030) supplemented with the Endothelial Cell Growth Kit–VEGF (ATCC, PCS-100-041). Cells were maintained at 37°C in a humidified incubator with 5% CO₂. HUVECs at passages below P6 were considered young, while those at passage P12 or higher were classified as aged. For treatment experiments, HUVECs were exposed to the following stimuli and inhibitors, either alone or in combination as indicated: heme (10 μM, Hemin, Sigma, Cat: 51280-5G), heat-killed E. coli (MOI: 20, ATCC, Cat: BAA197), STING inhibitor (2.5µM, C-176, MCE, Cat: HY-112906). Cells were treated for the indicated times to assess proliferation using Brdu staining, mitigation using wound healing assay, and angiogenic capacity using the tube formation assay. Cell senescence was determined via β-galactosidase (SA-β-Gal) activity using the Senescence β-Galactosidase Staining Kit (CST, Cat: 9860S) following the manufacturers’ protocols. Additionally, the expression of senescence markers was analyzed by qPCR assay, Western blot and immunostaining.

**Adeno-Associated Virus (AAV) packaging and in vivo Administration**

Recombinant adeno-associated virus (AAV) vectors expressing the human hemopexin (HPX) gene under a CMV promoter (pAAV-CMV-HPX, ABM, Cat: 23884101) were generated using a triple-plasmid system, including a packaging plasmid (pAAV2/8, Addgene Cat: 112864) and a helper plasmid (pAdDeltaF6, Addgene Cat: 112867). The pAAV-CMV-Blank vector served as a negative control. HEK293T cells were co-transfected with these plasmids using Lipofectamine 3000, and viral particles were harvested 72 hours later. HEK293T cells were co-transfected with the plasmids using Lipofectamine 3000, and viral particles were harvested 72 hours post-transfection. Viral purification and titering were performed according to published protocols. Mice received intravenous injections of AAV-HPX or control AAV at a single dose of 1 × 10¹¹ genome copies (GC) in 100 µL of sterile PBS via the tail vein. Two weeks post-AAV delivery, the cecal ligation and puncture (CLP) model of sepsis was performed as previously described. Transfection efficiency was confirmed by assessing HPX expression in liver tissues.

**Immunofluorescence, confocal imaging, and analysis**

At the indicated time points following CLP-induced sepsis, both sham and septic mice were anesthetized, and liver tissues were collected and fixed in 4% paraformaldehyde (PFA) for 12 hours at 4 °C, followed by immersion in 30% sucrose overnight. Samples were then embedded in OCT compound and sectioned at 10 μm thickness using a Leica CM3050S cryostat. For in vitro studies, HUVECs were cultured on chamber slides, washed with PBS, and fixed in 2% PFA for 20 minutes at room temperature. Both cryosections and HUVECs were washed three times with PBS, permeabilized with 0.5% Triton X-100 in PBS for 20 minutes at room temperature, and then blocked with 10% goat serum for 1 hour. Samples were incubated overnight at 4°C with the following primary antibodies: : In Situ Cell Death Detection Kit (TUNEL kit, Millipore Sigma, Cat#12156792910), anti-CD31 (endothelial marker, R&D, Cat #AF3628, 1:100), anti-p16 Cell Signaling Technology, Cat# #80772S, 1:100), anti-p21 (Cell Signaling Technology, Cat #2947S, 1:100), anti-p-STING (Cell Signaling Technology, Cat: 72971S, 1:100), anti-p-TBK1 (Cell Signaling Technology, Cat: 5483S, 1:100), anti-p-IRF3 (Cell Signaling Technology, Cat: 4947S, 1:100), and anti-STING (ABclonal, Cat# A21051, 1:100). Following primary incubation, sections and cells were washed once with PBST and twice with PBS, then incubated with appropriate Alexa Fluor–conjugated secondary antibodies for 1 hour at room temperature. After three final PBST washes, samples were mounted with DAPI-containing fluorescence mounting medium (Thermo Fisher Scientific, Cat# P36981) to stain nuclei. Confocal images were captured using a Leica TCS SP8 microscope and processed using ImageJ software (NIH) for quantitative and qualitative analysis.

**Protein Extraction and Western Blot Analysis**

Protein extraction from heart tissues and in vitro cultured HUVECs was performed using RIPA lysis buffer (Thermo Fisher Scientific, Cat: 89900) supplemented with protease and phosphatase inhibitors (Sigma-Aldrich, Cat: PPC2020). Protein concentrations were measured using a BCA protein assay kit (Thermo Fisher Scientific, Cat: 23223). For blue native PAGE (BN-PAGE), protein samples were prepared using the NativePAGE™ Sample Prep Kit (Invitrogen, Cat# BN2005) containing 10%DDM, along with NativePAGE™ 4× Sample Buffer (Invitrogen, Cat# BN2003). The 5% Coomassie G-250 sample additive (Invitrogen, Cat# BN2002) was added immediately prior to loading. Samples were resolved on NativePAGE™ Novex® 3–12% Bis-Tris gels (Invitrogen, Cat# NP0321BOX), and electrophoresis was performed using the XCell™ SureLock™ Mini-Cell system with the recommended Dark Blue and Light Blue cathode buffers. Following migration of the dye front to one-third of the gel, Dark Blue buffer was replaced with Light Blue buffer to reduce background interference during Western transfer. After electrophoresis, proteins were transferred to PVDF membranes using the XCell II™ Blot Module and NuPAGE® Transfer Buffer (Thermo Fisher Scientific, Cat# 88518, NP00061). Equal amounts of protein were separated by electrophoresis on Bis-Tris protein gels and transferred onto nitrocellulose membranes. Membranes were blocked with 5% BSA in TBS containing 0.5% Tween-20 (TBST) for 1 hour at room temperature, followed by incubation with specific primary antibodies overnight at 4°C. After washing with TBST, membranes were incubated with HRP-conjugated secondary antibodies (Cell Signaling Technology, Cat: 7074) for 1 hour at room temperature, followed by additional washes with TBST to remove unbound antibodies. Bands were visualized using the SuperSignal™ West Femto Maximum Sensitivity Substrate (Thermo Fisher Scientific, Cat: 34096), optimized for detecting low-abundance targets, and SuperSignal™ West Pico PLUS Chemiluminescent Substrate (Thermo Fisher Scientific, Cat: 34580) for higher-abundance targets. Imaging was conducted with the G:Box Chemi gel documentation system (GeneSys Version: 1.8.10.0), and band intensities were quantified using Image J software.

**Plasmid Transfection of STING and TBK1**

To increase the expression of STING and TBK1 in HUVECs, cells were seeded at ~60% confluence and transfected with either the human STING1 ORF cDNA expression plasmid containing a C-terminal HA tag or the human TBK1 ORF mammalian expression plasmid with a C-terminal Flag tag (Sino Biological, Cat# HG29810-CY and HG11023-CF, respectively). Transfection was performed using polyethylenimine (PEI, linear, MW 25,000, Polysciences, Cat# 23966) at a DNA:PEI ratio of 1:3. Briefly, plasmid DNA and PEI were each diluted in Opti-MEM (Gibco, Cat# 31985070), incubated at room temperature for 15 minutes to allow complex formation, and then added dropwise to the cells. After 4 hours, the transfection medium was replaced with complete endothelial growth medium. Cells were harvested 48 hours post-transfection for downstream applications.

**Antibodies**

The following primary and secondary antibodies were used in this study: Anti-STING (Cat: 13647S), anti-p-STING (Cat: 72971S), anti-TBK1 (Cat: 3504S), anti-p-TBK1 (Cat: 5483S), anti-IRF3 (Cat: 4302S), anti-p-IRF3 (Cat: 4947S), anti-p-IRF3 (Cat: 4947S), anti-p-IRF3 (Cat: 4947S), p21 (Cat: 2947S) were purchased from Cell Signaling Technology. Anti-p21(Cat: A22460), Anti-p16(Cat: A23882), anti-A-p53 (Cat: A19836), Anti- Hemopexin (HPX) (Cat: A5603), Anti-DDDDK-Tag (Cat: AE005) were purchased from Abclonal.

**RNA-seq and data analysis**

Total RNA was extracted from sham and septic mouse heart tissues using RNAzol RT reagent (Molecular Research Center, Cat# RN 190) according to the manufacturer’s protocol. RNA quality and concentration were assessed using an Agilent Bioanalyzer 2100, and samples with RNA integrity number (RIN) > 6.0 were used for library preparation. RNA quality control, library preparation, sequencing, and data analysis were performed by Genewiz (Azenta Life Sciences). Differential expression analysis was conducted using standard bioinformatics pipelines provided by the service provider. Gene Set Enrichment Analysis (GSEA) was performed using the GSEA software (Broad Institute) to assess pathway-level changes.

**Quantitative RT-PCR Analysis.**

Total RNA was isolated from mouse heart tissues and cultured cells using RNAzol RT reagent (Molecular Research Center, Cat: RN 190) according to the manufacturer’s protocol. cDNA was synthesized from 0.1–1 μg of total RNA using the High-Capacity cDNA Reverse Transcription Kit (Thermo Fisher Scientific, Cat: 4368814). Quantitative real-time PCR (qRT-PCR) was performed using SYBR Select Master Mix on a Bio-Rad CFX96 real-time PCR detection system. The following PCR primers were used: H-CDKN1A (forward, 5’-TGTCCGTCAGAACCCATGC-3’; reverse, 5’-AAAGTCGAAGTTCCATCGCTC-3’), M-Cdkn1a (forward, 5’-CCTGGTGATGTCCGACCTG-3’; reverse, 5’-CCATGAGCGCATCGCAATC-3’), H-CDKN2A (forward, 5’-GGAGGCCGATCCAGGTCAT-3’; reverse, 5’-ATGGTTACTGCCTCTGGTGC-3’), M-Cdkn2a (forward, 5’-CGCAGGTTCTTGGTCACTGT-3’; reverse, 5’-TGTTCACGAAAGCCAGAGCG-3’), M-Cxcl2 (forward, 5’-ATTCTGTGACCATCCCCTCAT-3’; reverse, 5’-TGTATGTGCCTCTGAACCCAC-3’), H-CXCL2 (forward, 5’-AGATCAATGTGACGGCAGGG-3’; reverse, 5’-TCTCTGCTCTAACACAGAGGGA-3’), H-CXCL1 (forward, 5’-TGCTGCCACTAATGCTGATGT-3’; reverse, 5’-CTCAGGAACCAATCTTTGCACT-3’), M-Il1a (forward, 5’-CGAAGACTACAGTTCTGCCATT-3’; reverse, 5’-GACGTTTCAGAGGTTCTCAGAG-3’), H-IL1A (forward, 5’-TGGTAGTAGCAACCAACGGGA-3’; reverse, 5’-ACTTTGATTGAGGGCGTCATTC-3’), H-SERPINE1 (forward, 5’-CCTGGGCACTTACAGGAAGG-3’; reverse, 5’-GGTCCGATTCGTCGTCAAATAAC-3’), H-FN1 (forward, 5’-CGGTGGCTGTCAGTCAAAG-3’; reverse, 5’-AAACCTCGGCTTCCTCCATAA-3’), H-Thbs1 (forward, 5’-AGACTCCGCATCGCAAAGG-3’; reverse, 5’-TCACCACGTTGTTGTCAAGGG-3’), M-Mmp8 (forward, 5’- TCTTCCTCCACACACAGCTTG-3’; reverse, 5’- CTGCAACCATCGTGGCATTC-3’), H-MMP8 (forward, 5’-TGCTCTTACTCCATGTGCAGA-3’; reverse, 5’-TCCAGGTAGTCCTGAACAGTTT-3’).

**Measurement of Heme Levels in plasma**

Blood samples were collected via cardiac puncture into tubes containing heparin and centrifuged at 3000g for 20 minutes to separate plasma. Plasma heme levels were measured using the Heme Assay Kit (Sigma-Aldrich, Cat:MAK316) according to the manufacturer’s instructions. Plasma hemopexin (HPX) levels were quantified using an ELISA kit (Abclonal, Cat: RK09237)

**Echocardiography**

M-mode tracings were used to measure left ventricular (LV) wall thickness, LV end-systolic diameter (LVESD), and LV end-diastolic diameter (LVEDD). Percent fractional shortening (%FS) and ejection fraction (EF) were calculated as previously described^44, 45^.

**Statistical Analysis**

All experiments were performed at least three independent times and representative data are shown. Statistical analysis between two groups were performed by an unpaired two-tailed Student’s t-test, while one-way ANOVA with Tukey’s post hoc test was used for multiple comparisons. A p-value less than 0.05 was considered statistically significant. Data are expressed as mean ± SD. Analyses were performed using GraphPad Prism 8.4.3 software.

**Figure S1. Sepsis induces cardiac senescence that correlates with disease severity**

(**A, B**) Immunofluorescent co-staining and quantification of the senescence marker p21 (red) and the endothelial marker CD31 (green) in heart sections from the indicated groups (n=6 mice/group). Scale bars, 25 μm. (**C, D**) Immunofluorescent co-staining and quantification of the senescence marker p16 (red) and the endothelial marker CD31 (green) in heart sections from the indicated groups (n=6 mice/group). Scale bars, 25μm. All data are presented as mean ± SD. *P < 0.05, **P < 0.01, ****P < 0.0001.

**Figure S2. Cardiac endothelial senescence impairs proliferation in sepsis.**

(**A**) Quantification of endothelial marker CD31 (green) in heart sections from sham and septic mice (n=6-10 mice/group). (**B, C**) Immunofluorescent co-staining and quantification of the proliferation marker Ki67 (red) and the endothelial marker CD31 (green) in heart sections from sham, septic survivors, and septic non-survivors (n=6-8 mice/group). Scale bars, 25 μm. (**D, E**) Immunofluorescent co-staining and quantification of TUNEL (red) and the endothelial marker CD31 (green) in heart sections from the indicated groups (n=6 mice/group). Scale bars, 25 μm. All data are presented as mean ± SD. *P < 0.05, **P < 0.01, ****P < 0.0001.

**Figure S3. Heme administration enhances cardiac endothelial senescence and impairs endothelial proliferation.**

(**A, B**) Immunofluorescent co-staining and quantification of the senescence marker p16 (red) and the endothelial marker CD31 (green) in heart sections from the indicated groups (n=6 mice/group). Scale bars, 25 μm. (**C, D**) Immunofluorescent co-staining and quantification of the proliferation marker Ki67 (red) and the endothelial marker CD31 (green) in heart sections from the indicated groups (n=6 mice/group). Scale bars, 50 μm. (**E, F**) Immunofluorescent co-staining and quantification of TUNEL (red) and the endothelial marker CD31 (green) in heart sections from the indicated groups (n=6 mice/group). Scale bars, 25 μm. All data are presented as mean ± SD. *P < 0.05, **P < 0.01, ****P < 0.0001.

**Figure S4. Heme exacerbates bacteria-induced impairment of endothelial reparative capacity.**

(**A, B**) Endothelial cell migration assay in HUVECs treated with heme, heat-killed *E. coli*, or both (n=6/group). (**C**) Endothelial tube formation assay under the same treatment conditions. (**D**) Quantification of total tube length and branching points in HUVECs (n=5/group). All data are presented as mean ± SD. Statistical analysis was performed using the unpaired two-tailed Student’s t-test: **P < 0.01, ***P < 0.001, ****P < 0.0001.

**Figure S5. Heme aggravates sepsis induced STING activation.**

(**A-C**) Immunofluorescent co-staining of p-STING, p-TBK1, p-IRF3 (red) and the endothelial marker CD31 (green) in heart sections from the indicated groups (n=6 mice/group). Scale bars, 20 μm.

**Figure S6. Heme aggravates bacterial infection induced STING activation.**

(**A-D**) Co-immunoprecipitation (Co-IP) assay indicate interaction between TBK1 and STING in HUVECs transfected with STING-HA and TBK1-Flag plasmids, followed by treatment with heme (10 µM), heat-killed *E. coli* (MOI:10), cGAMP (5 µg/mL), or their combinations for 1 hour. (**C, D**) Immunofluorescence staining of STING aggregates in HUVECs following treatment with heme, *E. coli*, cGAMP, or combinations. Scale bars, 20 μm. (**E-G**) Native PAGE Western blot analysis of HUVECs in groups as indicated. All data are presented as mean ± SD. *P < 0.05, **P < 0.01, ***P < 0.001, ****P < 0.0001.

**Figure S7. STING inhibition attenuates sepsis-induced cardiac endothelial senescence.**

(**A, B**) Immunofluorescent co-staining and quantification of the senescence marker p16 with the endothelial marker CD31 in heart tissues from sham and septic mice treated with a STING inhibitor or vehicle at 24 hours post-CLP (n=5-6 mice/group). Scale bars, 25 μm. (**C-F**) Immunofluorescent co-staining and quantification of senescence markers p16 or p21 with the endothelial marker CD31 in heart tissues from sham and septic mice treated with a STING inhibitor or vehicle at 14 days post-CLP (n=4-6 mice/group). Scale bars, 25 μm. All data are presented as mean ± SD. *P < 0.05, **P < 0.01, ***P < 0.001, ****P < 0.0001.

**Figure S8. STING inhibition restores endothelial STING activation in vivo.**

(**A-C**) Immunofluorescent co-staining of p-STING, p-TBK1, p-IRF3 (red) and the endothelial marker CD31 (green) in heart tissues from sham and septic mice treated with a STING inhibitor or vehicle at 24 hours post-CLP (n=5-6 mice/group). Scale bars: 20 μm.

**Figure S9. STING inhibition restores endothelial reparative function in vitro.**

(**A, B**) β-galactosidase staining and quantification of HUVECs treated with septic plasma, with or without STING inhibitor (C-176, 2.5 µM). (**C, D**) Immunofluorescent staining and quantification of senescence marker p21 in HUVECs treated with heme (10 µM) and heat-killed E. coli (MOI:10) for 6 hours , with or without STING inhibitor (n=6/group). Scale bars, 50 μm. (**E–H**) Endothelial proliferation (BrdU) and tube formation assays following treatment with combined heme and E. coli, with or without STING inhibitor. All data are presented as mean ± SD. *P < 0.05, **P < 0.01, ****P < 0.0001.

**Figure S10. STING inhibition reduced cardiac fibrotic remolding in chronic septic heart.**

(**A**) Picrosirius Red staining of heart tissues from sham and septic mice treated with a STING inhibitor or vehicle at 24 hours or 14 days post-CLP (n=6 mice/group). Scale bars: 100 μm.

**Figure S11. Increased hemopexin expression attenuates sepsis-induced cardiac endothelial senescence.**

(**A-D**) Immunostaining and quantification of the senescence markers p21 or p16 with endothelial cell markers in sham and septic heart tissues from AAV-HPX and AAV-CON mice at 24 hours post-CLP (n=6-7/group). Scale bars, 25 μm. All data are presented as mean ± SD. **P < 0.01, ****P < 0.0001.

**Figure S12. Increased hemopexin expression attenuates sepsis-induced cardiac endothelial senescence and enhances endothelial proliferative capacity.**

(**A, B**) Co-immunostaining and quantification of the senescence marker p16 with the endothelial marker CD31 in heart tissues from AAV-HPX and AAV-CON mice at 14 days post-sepsis. Scale bars, 50 μm. (**C, D**) Co-immunostaining and quantification of the cellular proliferation marker Ki67 with the endothelial marker CD31 in sham and septic heart tissues from AAV-HPX and AAV-CON mice at 24 hours post-sepsis. Scale bars, 50 μm. All data are presented as mean ± SD. *P < 0.05, **P < 0.01, ****P < 0.0001.

**Figure S13. Increased hemopexin expression enhances endothelial proliferative capacity and attenuates sepsis-induced cardiac endothelial fibrotic remolding.**

(**A, B**) Co-immunostaining and quantification of the cellular apoptosis marker TUNEL with the endothelial marker CD31 in sham and septic heart tissues from AAV-HPX and AAV-CON mice at 24 hours post-sepsis. Scale bars, 25 μm. (**C**) Picrosirius Red staining of heart tissues from sham and septic mice treated with AAV-HPX and AAV-CON at 14 days post-CLP (n=6 mice/group). Scale bars: 100 μm. All data are presented as mean ± SD. *P < 0.05, **P < 0.01.
